# Supplementary material for: Integration of variant annotations using deep set networks boosts rare variant association testing
Source: Nat Genet. 2024 Sep 25;56(10):2271–80. doi: 10.1038/s41588-024-01919-z (PMC11525182; doi:10.1038/s41588-024-01919-z)
Supplement: Supplementary file 1 — Supplementary Methods, Supplementary Results and Supplementary Figs. 1–11. [file 41588_2024_1919_MOESM1_ESM.pdf]

nature genetics

Supplementary information https://doi.org/10.1038/s41588-024-01919-z

Integration of variant annotations using deep set networks boosts rare variant association testing

In the format provided by the authors and unedited

Contents

1 Supplementary Methods **2**

1.1 DeepRVAT model . . . 2
1.1.1 Method overview . . . 2
1.1.2 Input and target data . . . 2
1.1.3 Gene impairment module . . . 2
1.1.4 Training procedure on seed genes . . . 2
1.1.5 Model implementation and hyperparameters . . . 3
1.1.6 Practical considerations for input data . . . 3
1.2 Relationship with prior work . . . 4
1.2.1 Principles of burden tests and SKAT . . . 4
1.2.2 Existing methods that handle more complex variant annotations . . . 4
1.3 Implementation of comparison partners . . . 5
1.3.1 Burden tests and SKAT . . . 5
1.3.2 STAAR . . . 5
1.3.3 Monti et al. . . . 6
1.3.4 REGENIE . . . 6
1.3.5 Combination of multiple p-values per gene and multiple testing correction . . . 6
1.3.6 Expected allele frequency filtering . . . 6
1.4 Rare-variant association testing with DeepRVAT . . . 6
1.4.1 CV scheme . . . 7
1.4.2 Leveraging pre-trained models and precomputed scores . . . 7
1.4.3 Integration into single-marker association testing frameworks . . . 7
1.5 Application to UK Biobank WES data . . . 7
1.5.1 Sequencing data preprocessing and quality control . . . 7
1.5.2 Custom sparse genotype data format . . . 8
1.5.3 Covariates and variant annotation . . . 8
1.5.4 Phenotype data . . . 9
1.5.5 DeepRVAT training and association testing . . . 9
1.5.6 Comparison with other UKBB RVAT studies . . . 10
1.5.7 Conditional association tests . . . 10
1.6 Phenotype prediction using DeepRVAT and alternative rare variant scores . . . 10
1.6.1 Problem statement . . . 10
1.6.2 Alternative burdens . . . 11
1.6.3 DeepRVAT gene impairment scores . . . 11
1.6.4 Phenotype predictor training and evaluation . . . 11
1.6.5 Phenotype predictor assessment . . . 12
1.7 Evaluation of feature importance . . . 12
1.7.1 Quantitative annotations . . . 12
1.7.2 Binary annotations . . . 13
1.7.3 Variant annotation groups . . . 13
1.8 Simulations . . . 13
1.8.1 Genotype data . . . 13
1.8.2 Overview of phenotype simulation . . . 13
1.8.3 Assessment of simulation results . . . 14
1.9 Practical recommendations for users . . . 14
1.9.1 Modes of usage . . . 14
1.9.2 Gene impairment module training . . . 14

2 Supplementary Results **15**

2.1 Model validation using simulated data . . . 15

3 Supplementary Figures **16**

4 Supplementary Table Captions **26**

1 Supplementary Methods

1.1 DeepRVAT model

1.1.1 Method overview

Model The DeepRVAT gene impairment module (Section 1.1.3) is trained as part of an end-to-end multi-phenotype prediction model. This model combines gene scores from the gene- and phenotype-agnostic impairment module using phenotype-specific weights to predict one or more phenotypes. During training, we restrict the genes used for phenotype prediction to a set of pre-defined *seed genes*, e.g. with known associations to the phenotypes of interest.

Applications Subsequently, using the trained DeepRVAT gene impairment module, impairment scores for all protein coding genes can be computed. The derived continuous scores can be leveraged in various downstream tasks, e.g., finding phenotype-associated genes through rare-variant association testing in large cohorts, such as the UK Biobank (UKBB). In addition, DeepRVAT gene impairment scores may be leveraged to augment conventional polygenic risk score (PRS)-based phenotype predictors by including the DeepRVAT scores to capture rare variant effects.

In the remainder of this section, we present a comprehensive description of the DeepRVAT model and how it is trained. Downstream analyses are described in subsequent sections.

1.1.2 Input and target data

Variant sets The input data for DeepRVAT consists of unordered sets of variants. The variant set for individual i and gene j is defined as

$$V_{ij} = \{(a_{kl})_{l=1,\dots,d} \mid \text{variant } k \text{ present in individual } i, \text{ gene } j\}, \tag{1}$$

where a_{kl} is the l-th annotation of variant k. Thus, V_{ij} is an unordered set of d-dimensional vectors, with each vector representing the d annotations of a given variant.

Targets As targets, we use a collection of P phenotypes, with the p-th phenotype of indivdidual i denoted by $y_i^{(p)}$. We used quantitative (continuous-valued) phenotypes in this study. DeepRVAT is trained in a multi-task model setup to predict all P phenotypes simultaneously (details below).

1.1.3 Gene impairment module

The DeepRVAT gene impairment module builds on a set neural network architecture to learn a trait-specific but gene-agnostic scoring function in a data-driven manner [1]. Specifically, the gene impairment module (denoted ψ in what follows) operates on sets of annotated variants and outputs a scalar score.

Architecture The variant set is first passed through a learnable submodule φ, which computes a *variant embedding* $\varphi(x)$ for each $x = (a_{k1}, \dots, a_{kd}) \in V_{ij}$. From the full set of variant embeddings, a fixed aggregation function f computes a *gene embedding*, then a second learnable submodule ρ computes a scalar *gene impairment score* $\psi(V_{ij})$ from this embedding. Finally, we pass the result through a sigmoid function. In full,

$$\psi(V_{ij}) = \sigma\left(\rho\left(f\left(\{\varphi(x)\}_{x \in V_{ij}}\right)\right)\right)$$

Both φ and ρ are multi-layer perceptrons (MLPs), i.e., feed-forward neural networks with multiple fully connected layers, and σ is the sigmoid (logistic) function.

Aggregation function The aggregation function f is required to be permutation-invariant, from which permutation invariance of the full phenotype prediction network follows. We also require that f produces outputs at a fixed dimension, independent of the number of elements in the variant set V_{ij}, since the MLP ρ requires fixed-dimensional input. Possible choices for f are the element-wise sum, product, or maximum, with output dimension d, or the `top2` function, with output dimension $2d$, containing the two largest values of each annotation.

1.1.4 Training procedure on seed genes

We describe here the full training procedure of the DeepRVAT model on training samples. Note that in practical scenarios, we employ a cross-validation scheme to restrict association testing to samples held out during model trainig; cf. Section 1.4.1.

Seed genes Training of the gene impairment module begins by selecting, for each training phenotype, a set of trait-specific *seed genes* from the set of all protein-coding genes. In this study, we base these on the results of alternative RVAT methods, specifically the "Burden/SKAT combined" method described in Section 1.3. We provide a pipeline to select seed genes by this method.

Training objective For a given individual i, we form variant sets V_{ij} for all seed genes j and compute the gene impairment score for gene j as $\psi(V_{ij})$. Following this, we estimate the p-th phenotype $y_i^{(p)}$ for individual i as a linear combination of the gene impairment scores and covariates:

$$\hat{y}_i^{(p)} = \boldsymbol{X}_i^T \boldsymbol{\alpha}^{(p)} + \sum_{j \in S^{(p)}} w_j^{(p)} \psi(V_{ij}),$$

where $\boldsymbol{X}_i$ is the vector of covariates for individual i, $\boldsymbol{\alpha}^{(p)}$ is a learnable vector of weights for the covariates, $S^{(p)}$ is the set of seed genes for phenotype p, and $w_j^{(p)}$ is a learnable weight for gene j.

Loss function We employed a simple multi-task learning objective across phenotypes, with the loss function given by the mean loss across all phenotypes. That is,

$$\mathcal{L}(y_i, \hat{y}_i) = \frac{1}{P} \sum_{p=1}^{P} \mathcal{L}(y_i^{(p)}, \hat{y}_i^{(p)}),$$

where again, P is the total number of phenotypes. The weights $\boldsymbol{\alpha}^{(p)}$ and $w_j^{(p)}$, as well as the parameters of ψ, are learned via backpropagation and minibatch gradient descent.

Parameter sharing The parameters of ψ are shared across all variants, genes, and phenotypes, while $\boldsymbol{\alpha}^{(p)}$ and $w_j^{(p)}$ are phenotype-specific. For the downstream analyses we describe in this work, the covariate and gene weights $\boldsymbol{\alpha}^{(p)}$ and $w_j^{(p)}$ are not needed – only the optimized gene impairment module ψ^* is used.

1.1.5 Model implementation and hyperparameters

We provide here the specific modeling setup as used to obtain the results reported in the manuscript.

Software versions All DeepRVAT models were implemented in PyTorch v1.13.1 and PyTorch-Lightning v1.5.10.

Training-validation split A data point for the DeepRVAT multi-phenotype model was given by an individual-phenotype pair. The target was that individual's value for phenotype p. The input was that individual's sets of annotated rare variants, one set for each seed gene. Prior to training, data points were shuffled, and a validation set consisting of 20% of individuals was selected at random for each phenotype. The validation set was randomly chosen on a per-phenotype basis, meaning it could differ across phenotypes.

Architecture hyperparameters For the variant embedding φ, we used a two-layer MLP with width 20. We used an MLP with two hidden layers of width 10 for ρ. In both networks, leaky ReLUs with negative slope 0.01 were used as the activation functions.

Training hyperparamters During training, we used the mean-squared-error (MSE) loss and the AdamW optimizer [2] with learning rate 0.001. The batch size was 1024. During training, the MSE loss was monitored every epoch on the validation set, and the training checkpoint with lowest validation MSE loss was retained. Training proceeded for a minimum of 50 and a maximum of 1,000 epochs, with early stopping implemented by the PyTorch-Lighting `EarlyStopping` callback with metric the validation MSE, `patience` 3 and `min_delta` 1e-5.

Ensembling DeepRVAT models can be ensembled, with the gene impairment score computed as the mean of all trained models. For each trained model, we randomly sampled a new training and validation dataset as in Section 1.1.5. We used six models in our ensemble, finding improvements in performance saturated after this. Since the DeepRVAT model is comparatively small, we trained all six models simultaneously on a single GPU using GNU `parallel` ([3]).

1.1.6 Practical considerations for input data

Variant and annotation data Input data preparation begins with computing all annotation scores for all variants present in the analyzed cohort if they lie in the genomic regions to be considered (e.g., all protein-coding genes). Next, using the cohort genotype data together with genome annotations, the variant set for each individual–gene combination must be determined to obtain matrices of `annotations x variants`. These matrices for each individual and each gene are then combined to obtain the final input tensor. In our practical implementation of DeepRVAT, we achieved the greatest computational efficiency by forming a padded tensor with dimensions of `individuals x genes x annotations x variants`, where, in a given minibatch, the final dimension is padded to the largest number of variants in any individual/gene combination. For model training, the input tensor only comprises the seed genes, with one input tensor used per phenotype.

Phenotypes and covariates Besides the variant information, DeepRVAT training and association testing requires the true phenotype values (`individuals x phenotypes`) and the covariates (`individuals x covariates`) to be considered (e.g., age, sex, genetic principal components).

1.2 Relationship with prior work

Broadly, rare variant association testing methods can be categorized into burden tests (also called collapsing tests) and variance-component tests. To increase power, both kinds of tests require a grouping of variants into variant sets (e.g., genes), and incorporate filtering to include only putative causal variants (e.g., pLOF and/or missense variants). They also require applying a predetermined weighting function to individual variants.

1.2.1 Principles of burden tests and SKAT

The most widely-used methods remain conventional burden tests and the sequence kernel association test (SKAT). Although these models do not conduct any specific modeling to handle more complex annotations, they remain canonical baselines and underpin many more advanced choices [4, 5].

Burden tests Burden tests [6] collapse variant weights by, e.g., sum or a binary indicator (presence/absence of a qualifying variant), into a single score per sample and variant set. This score is then tested for association with traits. This simple form of burden test, which is also the most commonly applied, assumes all filtered variants are causal and have the same direction of effect.

Variance component tests Variance component tests such as SKAT [7] treat variant effects as random effects and assume that the effects in a given variant set are normally distributed around zero. They test against the null hypothesis that the distribution of effects is a delta distribution at zero. Thus, these tests can handle situations where variants of opposite effects and non-causal variants are present.

SKAT and SKAT-O SKAT is a specific type of variance component test for association, utilizing a kernel matrix to estimate the similarity between individuals based on their genotype. More specifically, the kernel in SKAT is given by $K = GWWG^T$, where G is the $n \times p$ genotype matrix (with n the number of samples and p the number of variants) and $W = diag(w_1, \ldots, w_p)$ is a specified vector of variant weights. An extension of SKAT, SKAT-O [8], interpolates between SKAT and burden tests, based on the observation that a burden test can be viewed as a special type of kernel test. Annotations enter both SKAT and burden tests as part of the preprocessing of the input data. Variants are typically filtered by allele frequency and pLOF or missense annotations (see also [9, 10] for guidelines and examples). This can comprise simply including all variants of one type (e.g., missense or pLOF [4]). In other schemes, additional annotations indicating the degree or probability of deleteriousness are taken into account (e.g., [5]). Also, instead of commonly used variant weighting based on variant MAF only, single or combinations of annotation scores might be used to weight variants in both burden tests and SKAT [11].

1.2.2 Existing methods that handle more complex variant annotations

Building on SKAT and burden tests, there has been a history of model refinements and advances to allow for making use of more complex annotations. In the following, we review the most relevant developments and their relationship to DeepRVAT (see also Supplementary Table. 1).

Hierarchical mixed-effect models An early example of a method that utilizes functional annotations in a data-driven manner is MiST [12], which introduces a hierarchical mixed-effects model for RVAT. Later, BATI [13] has been proposed as a Bayesian generalization of this model, which uses Integrated Nested Laplace Approximation to improve robustness. While being capable of considering different variant characteristics jointly, both models are linear and learn the relevance of annotations indivdually for each gene, which in practice limits the number and complexity of annotations that can be considered.

Meta-models Another emerging group of methods, which we classify here as *meta-models*, perform post-hoc meta-analysis of multiple tests, which can comprise different variant filters (e.g., pLOF or missense variants), test types (e.g., burden test or SKAT), and/or weightings based on single annotation scores or groups of related annotations [14, 15, 16, 11].

Kernel methods for multiple annotations For example, Konigorski et al. [16] and Monti et al. [11] proposed linear mixed-effect models that can learn variant effects based on variant weights or on annotations directly. They also developed kernels which allow for user-specified mappings of variants into a feature space, with variant effects modeled linearly based on their representations in feature space. Roughly, then, variant effects are modeled based on their similarity in feature space. Practically, multiple tests for different types of variants are used, each of which defines a weighting or variant similarity kernel based on a set of related annotations.

STAAR The STAAR method [15] takes a different perspective on combining multiple annotations into a single test, by first testing for association between each single annotation and a trait, then using statistical methods (namely, the Cauchy combination test [17, 18]) to combine the p-values from these individual tests into a single score. Additionally, the authors use PCA to reduce the total number of annotations tested and the empirical cumulative distribution of annotations to estimate the probability of a variant being causal.

Limitations of existing methods While STAAR, the methods of Konigorski et al. and Monti et al. have been shown to be scalable to handle biobank-scale datasets, these meta-models are limited to test individual variant annotations or groups of related annotations in isolation rather than jointly. They are also not suitable for computing a single gene score that can be used in related tasks, such as phenotype prediction.

Novel characteristics of DeepRVAT These existing RVAT methods and our method share some conceptual advances over burden tests and SKAT, including data adaptivity, the usage of functional annotations—including those derived from predictions of deep neural networks—and the mapping of variants into feature space. However, to the best of our knowledge, our approach is the first to learn, in a data-driven manner, nonlinear feature mappings from sequencing studies, and also the first to directly incorporate deep neural networks into an RVAT framework. Furthermore, DeepRVAT contrasts with these methods in that it learns from signal across multiple genes during training time, applying these jointly learned variant representations to other genes in the association testing phase. Finally, DeepRVAT is a single model that is directly applicable to phenotype prediction, while methods based on meta-models require a scheme to combine predictions across multiple models, and have not been considered for this task.

1.3 Implementation of comparison partners

We compared DeepRVAT to burden tests, SKAT, STAAR, and Monti et al. [11], and, for binary traits, REGENIE [19] with default settings. In the following, we provide details on the implementation of these methods.

1.3.1 Burden tests and SKAT

Test types, variant annotation We implemented burden and SKAT tests following [4], using the score test from the `SEAK` package[1] (v0.4.3) [16, 11]. For quantitative (resp. binary) phenotypes, a null model was computed using the `ScoreTestNoK` (resp. `ScoreTestLogit`) class, followed by calling the `pv_alt_model()` method to compute a p-value.

All combinations of burden and SKAT tests restricted to either pLOF or missense variants were carried out, giving four method/variant type combinations. The pLOF variant category comprised all variants annotated as stop gained, start lost, splice donor, splice acceptor, stop lost or frameshift by Ensembl Variant Effect Predictor (VEP) [20, 5]. Annotation of missense variants was also carried out using VEP. Each method/variant type combination was carried out for all protein-coding genes. To reduce the data sparsity due to ultra-rare variants in SKAT tests, variants with a with minor allele count (MAC) ≤ 10 were collapsed and then tested together with all other variants with MAC > 10 as described by [21]. Due to computational constraints, we skipped genes with over 5000 markers, impacting only one gene (Titin) for missense variant tests.

Variant weights The weight for each variant v_j was $w_j = \mathrm{Beta}(\mathrm{MAF}(v_j); 1, 25)$ where $\mathrm{Beta}(\cdot; 1, 25)$ denotes the Beta density function with parameters $(1, 25)$, which upweights rarer variants.

Combination test, multiple testing correction In addition to the four individual tests, we created a combination test (*Burden/SKAT combined*) using the full set of p-values from all four individual tests. The resulting significant gene-trait associations from this combined test were subsequently used as seed genes for training DeepRVAT (see Sec. 1.1 below).

1.3.2 STAAR

Software STAAR tests were implemented in R using the `STAAR` package provided by the authors[2] and following the vignette provided in the package, as well as the procedures in the original publication [15].

Variant annotation STAAR requires annotations of variants, and to insure optimal comparability with DeepRVAT, the same annotations as described below in Sec. 1.5 were used. As required for the STAAR procedure, each annotation a_{jk} for variant j was PHRED-scaled according to the formula

$$a_{jk}^{\mathrm{PHRED}} = -10 \log_{10}(1 - q_{jk}),$$

where q_{jk} represents the quantile of a_{jk} when considering the distribution of annotation k across all variants in the dataset.

[1] `https://github.com/HealthML/seak`

[2] `https://github.com/xihaoli/STAAR`

Variant groups, multiple testing correction Following [15], STAAR p-values were computed for five variant groups, namely (1) putative loss-of-function (stop gain, stop loss and splice), (2) missense, (3) disruptive missense, (4) putative loss-of-function and disruptive missense, and (5) synonymous variants. We defined disruptive missense variants to be those that were predicted to be both "deleterious" by SIFT [22] and "probably damaging" by PolyPhen2 [23]. This yielded five p-values per gene.

1.3.3 Monti et al.

We conducted various collapsing and kernel tests following the methodology described by Monti et al.[11]. We used the same annotations, variant weight thresholds, and variant kernel architectures as outlined in their study. Annotation scores were obtained according to the details provided in Supplementary Table 3 and Sec. 1.5.

Test types Specifically, we performed the following tests for different types of variants:

- Protein loss of function: Gene-based collapsing test.
- Missense variants: Weighted gene-based variant collapsing and kernel-based association tests. We used SIFT and PolyPhen2 scores for variant weighting and a local (amino-acid level) collapsing kernel to aggregate variants.
- Splicing: Weighted gene-based variant collapsing and kernel-based association tests. We used SpliceAI delta scores for variant weighting together with a linear kernel.
- RBP-binding: We employed a weighted, kernel-based association test using DeepRiPe predictions for six RBPs (QKI, MBNL1, TARDBP, ELAVL1, KHDRBS1, and HNRNPD). Following the approach described in Monti et al. (2022), the kernel matrix was generated using the Cholesky decomposition of the element-wise product of $Q \circ R$, where Q represents the similarity of variants based on their six DeepRiPe scores and R represents the similarity based on variant position.

Combination of p-values For all the aforementioned tests, we utilized the score test from SEAK. In the case of missense and splicing tests, if either the collapsing or kernel-based association test yielded nominal significance ($p < 0.01$), we performed joint testing with pLOF variants. The p-values from these tests were integrated using the Cauchy combination method, as described in [11]. In total, we obtained six p-values per gene.

1.3.4 REGENIE

Test types Burden and SKAT tests were run using both missense and pLOF masks, yielding four combinations as in 1.3.1. For burden tests, we used the default REGENIE strategy of collapsing variants to gene level using the maximum number of ALT alleles across sites. We used the approximate Firth likelihood ratio test for p-values less than 0.01.

Variant weights As in 1.3.1, weights for SKAT tests were computed using the $\mathrm{Beta}(\cdot; 1, 25)$ function.

1.3.5 Combination of multiple p-values per gene and multiple testing correction

The methods Burden/SKAT combined, Monti et al., and STAAR yielded multiple p-values per gene. These were aggregated at the gene level by adjusting for multiple testing via the Bonferroni procedure. We retained only the smallest p-value per gene for subsequent analyses. To account for multiple testing across all 19,388 tested genes, we applied Bonferroni correction, setting the genome-wide significance threshold at $\alpha = 0.05/19388 = 2.510^{-7}$.

1.3.6 Expected allele frequency filtering

Since burden and variance-component tests (that is, all comparison partners listed above) use variant filters to define qualifying variants (e.g., pLOF, missense, or disruptive missense), we followed the methodology of [4] to improve the reliability of the tests. Specifically, we restricted testing to genes that passed an expected allele frequency (EAF) filter of at least 50. The EAF is defined as $\mathrm{CAF} \cdot n$, where CAF is the cumulative allele frequency (the sum of allele frequencies of all qualifying variants j in the gene) and n is the cohort size for quantitative traits or the number of cases for binary traits.

1.4 Rare-variant association testing with DeepRVAT

Applying DeepRVAT can include training and association testing, or association testing with pretrained models. To avoid overfitting to a given dataset and obtaining inflated p-values, training and association testing with DeepRVAT is carried out in a cross-validation (CV) scheme. Additionally, DeepRVAT gene impairment scores can be seamlessly integrated into any framework for single-marker association testing. We give details on each of these points in this section.

1.4.1 CV scheme

Training The dataset D is first used for seed gene discovery using the *Burden/SKAT combined* method described in Section 1.3.1. Next, D is partitioned across samples into K equally-sized subsets, $D_1, \ldots, D_K$. For each $k = 1, \ldots, K$, a gene impairment module ψ_k^* is fit on the training set $\hat{D}_k = D \setminus D_k$ and discovered seed genes. Finally, a gene impairment score $h_{ij} = \psi_k^*(V_{ij})$ is computed for each sample i in the test set D_k and each gene j. This yields a dataset of the same sample size as D which can be used for association testing, but avoiding overfitting by computing gene impairment scores on samples not used during training of the given model.

Association testing Once all gene impairment scores have been computed, we follow the standard burden-testing approach and fit a linear model for each gene j:

$$g\left(y_i^{(p)}\right) = \boldsymbol{X}_i \boldsymbol{\gamma}^{(p)} + \beta_j^{(p)} h_{ij} + \varepsilon.$$

Here, g is an appropriate link function, $y_i^{(p)}$ is the value for target phenotype p in sample i, $\boldsymbol{X}_i$ is the vector of covariates for sample i, $\boldsymbol{\gamma}^{(p)}$ and $\beta_j^{(p)}$ are learned weights, h_{ij} is the gene impairment score for gene j of sample i, and ε is unexplained variance. We are interested in the effect size $\beta_j^{(p)}$ and its p-value.

1.4.2 Leveraging pre-trained models and precomputed scores

In addition to a full training and association testing pipeline, the DeepRVAT package contains pipelines for:

Pretrained models To perform association testing (as in Section 1.4), gene impairment scores h_{ij} are calculated for all sample/gene pairs of interest using the pre-trained DeepRVAT gene impairment modules across all repeats, as detailed in Section 1.4.1. These modules require variant annotation vectors (a_{kl}) with the same d annotations used during DeepRVAT gene impairment module training. Once the gene impairment scores h_{ij} are computed for the desired gene/individual pairs, they can be used to discover associations with quantitative or binary traits of interest, resulting in one p-value per tested trait and DeepRVAT repeat.

Precomputed gene impairment scores For UK Biobank data, we have returned DeepRVAT gene impairment scores computed as described in Section 1.5.5 below. This allows users to carry out new rare variant association studies without the necessity of directly working with genetic variant data or implementing the logic to compute burden scores with the correct model from the CV scheme in the previous subsection.

1.4.3 Integration into single-marker association testing frameworks

Since DeepRVAT provides a single score per gene and sample, it can be seamlessly integrated into any tool that carries out single-marker association testing with genotype dosages.

Conversion to BGEN Practically, we implement this by providing a script that uses the `bgen` package[3] v1.6.1 to convert the `samples` $\times$ `genes` matrix of DeepRVAT scores to a BGEN file [24]. The DeepRVAT gene impairment score h_{ij} is stored as the probability $p_{ij} = (h_{ij}, 0, 1 - h_{ij})$ of homozygous alternate, heterozygous, and and homozygous reference alleles, resp., so that the dosage $d_{ij} = 2h_{ij}$. Since $0 < h_{ij} < 1$, this puts DeepRVAT scores within the usual range of $[0, 2]$ for genotype dosages.

DeepRVAT+REGENIE The BGEN file can be used for single-marker association testing with REGENIE; for more details, see Section 1.5.5 below. However, any other single-marker association testing framework could also be used, as the BGEN file we output can be readily converted to any other standard genetic format.

1.5 Application to UK Biobank WES data

1.5.1 Sequencing data preprocessing and quality control

Exome sequencing for model training and benchmarking Whole-exome sequencing (+100 bp overhang) was performed on 200,633 participants from the UK Biobank [25], for which the methods have been described in the earlier release of data from approximately 50,000 individuals [26]. We will refer to this as the *UKBB 200k WES* dataset in what follows.

Full exome-sequencing cohort Whole-exome sequencing was also carried out on a larger cohort of UK Biobank participants. This resulted in a dataset totaling 469,779 participants, which we will refer to as the *UKBB 470k WES* dataset.

[3] https://github.com/jeremymcrae/bgen

Variant data and QC For both cohorts above, variant calling data was downloaded from the UK Biobank as project-level VCF (pVCF) files. Since the dataset had not been subjected to variant- or sample-level filtering prior to release by the UK Biobank, we applied additional quality control(QC) following [26]. All filtering steps were performed using bcftools v1.10.2 [27]. We required a minimum read depth of 7 for SNPs and 10 for indels. After read-depth filtering, only variant sites with at least one homozygous variant genotype or where at least one sample per site had an allelic balance ratio greater than 15% for SNPs and 10% for indels were retained. Indels were left-aligned and normalized, and multi-allelic variants were represented as multiple bi-allelic variants. Finally, we removed duplicate variants and filtered for variants where the fraction of missing genotypes was $< 10\%$ and the Hardy-Weinberg equilibrium p-value was $> 10^{-15}$. In total, our filtering criteria removed 5,336,543 out of 17,981,684 initial variants (29.67%). After excluding sex chromosomal variants, we ended up with 12,417,590 variants. Additionally, we filtered out individuals with $> 10\%$ missing genotype rate, which did not result in any individuals being dropped. Additionally, following the analysis best practices recommended by UK Biobank, we applied an additional coverage filter, requiring that at least 90% of all genotypes for a given variant have a read depth of at least 10. After these filters and additionally dropping all participants who had withdrawn from the study, we obtained datasets with 200,583 individuals and 12,704,497 variants (*UKBB 200k WES*), resp. 469,382 individuals and 26,141,967 variants (*UKBB 470k WES*).

1.5.2 Custom sparse genotype data format

Raw data After QC, we required the data to be stored in a format that allowed for fast, repeated loading over multiple epochs of DeepRVAT model training. To meet our needs, we constructed a custom sparse genotype data format as follows.

Genotype extraction After using the bcftools `norm` function for normalization and left alignment, we obtained BCF output files. Next, we extracted triplets of variant metadata, sample ID, and genotype from the BCF files, where the genotype was encoded as 1 (heterozygous) or 2 (homozygous-alternative), thereby generating a set of gzipped TSV files of 71 GB total, with one line for every variant present in a sample. Homozygous-reference genotypes were ignored for the purposes of these files. Following this, each unique variant was assigned an integer ID.

Custom HDF5 format As the last step, we created our custom sparse dataset in Hierarchical Data Format 5 (HDF5 v1.10.6). Genotype data was encoded as two equal-sized matrices, a variant and a genotype matrix, with the rows corresponding to individuals and the number of columns equal to the maximum number of variants found in any sample (60,247). Each row of the variant matrix provided the IDs of variants present in the corresponding individual, while the row of the genotype matrix provided the corresponding genotypes (1 or 2). Unnecessary elements at the end of each row were padded with -1. Sample IDs corresponding to the rows of the matrices were stored as an additional sample vector. Details on variants such as position and chromosome, as well as reference and alternative allele, were provided as a variants dataframe in Apache Parquet format. In total, the HDF5 dataset had a storage size of approximately 100 GB, compared to multiple terabytes for the original pVCF files.

1.5.3 Covariates and variant annotation

Covariates We retrieved genetic sex, sample age, age^2, age·sex and the first 20 genetic principle components (PCs) directly from UK Biobank (Supplementary Table 5). All of these covariates were included in association testing and when training DeepRVAT.

Variant-to-gene assignments Variants were assigned to genes using those protein-coding genes genes and their exons marked as golden in the merged Ensembl/HAVANA genome annotations (GENCODE release 38). We assigned a variant to a gene if it was located at most 300 bp from an exon of that gene. Multiple gene assignments were possible for a single variant.

Annotations The full collection of variant annotations used and their sources is provided in Supplementary Table 3. Here, we give details on processing for those annotations which were not used directly in the form output by the source.

MAF MAF values for variants were first replaced with the maximum of the MAF in the UK Biobank cohort and in gnomAD release 3.0 (non-Finnish European population). Following [6], The MAF p_j of each variant j was then transformed according to the formula $[p_j(1-p_j)]^{-\frac{1}{2}}$ for use in modeling.

VEP consequences We used 11 moderate- and high-impact consequences from VEP. These were encoded for each variant as multi-hot vectors, with a 1 in the corresponding column indicating that a consequence was predicted, and 0 if not.

DeepSEA DeepSEA predicts 919 different predicted variant effects on transcription factor binding, DNase I sensitivities, and histone marks in various cell types [28]. To improve model fitting and avoid overfitting, we performed principal components (PC) analysis and restricted to the first 6 PCs, explaining approximately 58% of variance.

SpliceAI SpliceAI provides four "delta scores" indicating a variant's predicted effect on cryptic splicing (acceptor gain, acceptor loss, donor gain, and donor loss) [29]. We computed the maximum of these four scores and used it as a single annotation.

AbSplice AbSplice-DNA predicts variant effects on aberrant splicing across 49 human tissues [30]. We computed the maximum predicted effect across tissues and used this as a single annotation.

DeepRiPe DeepRiPe characterizes in vivo RNA binding protein (RBP) binding preferences [31]. As in [11], we predicted effects of genetic variants on the binding of six RBPs over three cell lines using the pre-trained models from [32].

Annotation vectors Given the full set of annotations and the transformations described above, vectors representing variants had 34 dimensions in total.

MAF thresholds For DeepRVAT training (see 1.5.5), we used variants with MAF $< 1\%$. For association testing with all methods, we designated rare variants as having MAF $< 0.1\%$. Additionally, for both training and association testing, we restricted to variants with PHRED-scaled CADD value > 5.

1.5.4 Phenotype data

All phenotype data was obtained directly from UK Biobank, with the exception of waist-to-hip ratio (WHR), which was computed as the ratio of UKBB data field 48 to data field 49 and corrected for body-mass index (BMI) by regressing out the corresponding data field 21001.

Quantitative traits In case multiple instances of the phenotype were available, we chose the instance with the largest number of individuals having a measurement. Phenotype values were quantile transformed to match their empirical distributions to a standard normal distribution. For individuals with reported Statin usage, we adjusted Cholesterol (30690) by dividing by 0.8 and LDL-direct (30780) by dividing by 0.7, following [33, 34]. Statins considered were obtained from [35, Supplementary Table 1] and matched to UKBB treatment codes (20003). Supplementary Table 4 provides an overview of the full set of quantitative phenotypes and their corresponding data fields in UK Biobank.

Binary traits Binary traits were extracted using the definitions from [36, Supplementary Table 1]. Phenotype values were set to 1 for an individual if any "Matching Code" was found for the corresponding "Field" in that table. Otherwise they were set to 0. The exception is if the corresponding "Exclude" value was 1: In this case, the phenotype was set to `NA`.

1.5.5 DeepRVAT training and association testing

Subselected cohorts Since the various methods used for benchmarking control for sample relatedness and population structure differently, or not at all, we retained only unrelated individuals of European genetic ancestry from the *UKBB 200k WES* dataset for DeepRVAT model training and benchmarking against alternative RVAT methods. We used the `ukb_gen_samples_to_remove` function of the ukbtools R-package (v0.11.3, [37]) together with pre-computed relatedness scores (see UK Biobank Resource 668) to remove closely related individuals, keeping only one representative of groups that are related to the 3rd degree or less. Individuals of European ancestry were identified using UK Biobank data field 22006 (termed 'Caucasian'). This filtering resulted in a dataset (called *UKBB 200k unrelated European ancestry* below) of 161,822 individuals. For testing the integration of DeepRVAT with REGENIE (Fig. 3a), we additionally used all 167,214 individuals of European genetic ancestry.

Training and association testing Seed gene discovery and DeepRVAT training were carried out on the *UKBB 200k unrelated European ancestry* dataset. The training phenotypes were chosen among those for which at least 3 seed genes were available, yielding 21 phenotypes for training and 13 held out for association testing with pretrained models. Training was done according to the CV scheme described in Section 1.4.1, which also yielded gene impairment scores for all genes in this subset of the cohort. An ensemble consisting of all 30 models from the CV step (6 ensemble models from 5 training folds) was used to compute gene impairment scores for the remaining 307,560 individuals from the *UKBB 470k WES* cohort.

EAF filter Since DeepRVAT considers both high- *and* moderate-impact variants, the EAF filter as described in 1.3 did not have any effect on the set of genes included in association testing.

Association testing with SEAK For the method denoted *DeepRVAT*, association testing was carried out using the score test from SEAK v0.4.3, similarly to Section 1.3.1.

Association testing with REGENIE Association testing for the method *DeepRVAT+REGENIE* was carried out with REGENIE v3.4.1. REGENIE is run in two steps: In step 1, a set of phenotype-specific predictors is built from genetic markers using a two-level ridge regression approach, and in step 2, association testing with the markers of interest is carried out.

Following the REGENIE documentation, for step 1, we selected approximately 500k (precisely, 483,446) imputed SNPs from UKBB data field 22828, which were imputed using a combined panel from the Haplotype Reference Consortium [38] and the UK10K haplotype resource [39]. To do so, we used the following filtering in PLINK v2.00a2LM [40]: MAF < 0.06, MAC > 100, genotyping rate > 0.99, Hardy-Weinberg p-value $\geq 10^{-15}$, and sample missingness < 0.1. Additionally, we pruned SNPs with a pairwise linkage disequilibrium r^2 threshold of 0.9, using a window size of 1,000 and a step size of 100. Step 1 of REGENIE was then run with a block size of 1,000.

Step 2 of REGENIE was run on DeepRVAT gene impairment scores for each gene, derived as described in 1.4.3. For quantitative traits, the default options of REGENIE were used. For binary traits, we used the approximate Firth likelihood ratio test with a p-value threshold of 0.01.

Multiple testing correction To account for multiple testing across all 19,388 tested genes, we applied Bonferroni correction, setting the genome-wide significance threshold at $\alpha = 0.05/19388 = 2.510^{-7}$.

1.5.6 Comparison with other UKBB RVAT studies

We compared to gene-trait associations from two studies [4, 5] on larger WES cohorts from UK Biobank (454,787 and 394,841 individuals, respectively). We counted as a discovery any association that was considered significant according to the methodology of the study. For [4], we included tests using missense or pLOF burdens and genes that met the EAF filter as described in 1.3.6.

Assessment of replication To compute replication for quantitative traits in Fig. 2a,g and Fig. 4a, we first computed the set of discoveries from the studies mentioned above as

$$D^{(C)} = \{(g, p) \mid \text{ gene } g \text{ significantly associated to phenotype } p\}$$

Next, for each method m, we collected gene-trait associations (g, p) for all genes g and phenotypes p, and ranked them by p-value, resulting in a list $(g_1, p_1), \ldots, (g_N, p_N)$. The discoveries of rank m or less were then $D_m = \{(g_q, p_q) \mid 1 \leq q \leq m\}$, and the replication rate at rank m was defined as $|D_m \cap D^{(C)}|$.

Novel DeepRVAT discoveries on binary traits All significant discoveries made by DeepRVAT on binary traits in the analysis of the *UKBB 470K* dataset were compared to [36], where trait definitions matched precisely. Any gene-trait combination found in that study was considered known from previous UKBB RVAT studies. Additionally, the comparison to [4, 5], where trait definitions from this study and the comparison study could not be automatically mapped, was carried out by manual curation. If the gene from a gene-trait discovery by DeepRVAT was significantly associated with any related phenotype in the comparison studies, this discovery was considered known from previous UKBB RVAT studies. Those that were unknown from any of the three studies were included in Table 1.

1.5.7 Conditional association tests

For associations that were significant after multiple testing correction, we conducted conditional association tests using GWAS summary statistics from the Pan-UK Biobank [41]. Independently associated variants were identified from GWAS summary statistics through LD-based clumping using PLINK v1.9 [40] with default parameters, restricting to associations with a *p*-value $< 10^{-7}$ and MAF $> 1\%$. If a binary trait definition used in this study did not exactly match a single GWAS from Pan-UK Biobank, we combined *p*-values from all relevant GWAS that covered parts of the trait definition before performing clumping. For association testing with SEAK (i.e., *DeepRVAT*), independently associated variants within 500kb around the gene boundaries were incorporated as covariates in the conditional analysis. For association testing with REGENIE (i.e., *DeepRVAT+REGENIE*), all variants independently associated with a specific trait were considered for all genes.

1.6 Phenotype prediction using DeepRVAT and alternative rare variant scores

1.6.1 Problem statement

Tasks Here, we combine conventional common variant polygenic risk scores (PRS) with rare variant gene burdens, comparing the performance of DeepRVAT gene burden scores with alternative scores. Two separate problems were addressed: the prediction of raw phenotype values, and prediction of high-risk individuals. For the latter, we separately assessed two binary high-risk individual stratification tasks: (1) lowest 0.01-th quantile (ranked by phenotype value) vs. rest, and (2) highest 0.01-th quantile vs. rest.

Dataset Training and evaluation of the regression models was done on two disjoint data sets, restricting to unrelated individuals of European ancestry. A total of 154,966 (from *UKBB 200k WES*) and 224,817 individuals (from *UKBB 470k WES*, not found in *UKBB 200k WES*) were used for training and evaluation, respectively.

PRS computation Common PRS variants and effect sizes were all obtained from the Polygenic Score (PGS) Catalog [42] using the study from [43]. The catalog numbers of each common variant PRS are listed in Supplementary Table 4.

1.6.2 Alternative burdens

Gene discovery The training individuals were used for gene discovery using the method "Burden/SKAT combined" described in Section 1.3.1. Retaining associations at FWER < 0.05 resulted in a set of genes $G_b^{(p)}$ for phenotype p to use in the baseline prediction models.

Burden scores Subsequently, for each sample i (training and evaluation), each gene $j \in G_b^{(p)}$, and each annotation k considered, burdens s_{ij}^k were computed by taking the maximum of annotation l, i.e.,

$$s_{ij}^l = \max_{k \in W_{ij}} a_{kl}, \tag{2}$$

where W_{ij} denotes the set of all variants k in sample i and gene j, and a_{kl} is the l-th annotation of variant k. For the SIFT score, the minimum value was used since a score of 0 indicates the strongest effect.

1.6.3 DeepRVAT gene impairment scores

Gene impairment scores Computation of gene impairment scores was carried out analogously to Section 1.5.5. The same pretrained gene impairment modules as in that section were used, with scores computed according to the CV scheme on the training set. For the evaluation set, we averaged the scores of the 30 gene impairment module versions obtained during CV training.

Gene discovery Gene discovery was conducted across all 33 traits of interest[4] exclusively on training samples, following the method outlined in Section 1.4.1, using the gene impairment scores obtained as described in the previous paragraph. This yielded a set of trait-associated genes $G_d^{(p)}$ with an FWER < 0.05.

1.6.4 Phenotype predictor training and evaluation

For simplicity, we describe models for predicting raw phenotype values; prediction of extreme values is analogous, with logistic regression on the binary target replacing linear regression.

Baseline As a baseline phenotype predictor, we consider a regression model where the explanatory variables comprise covariates (age, sex, the first 20 genetic PCs) and the common variant PRS score:

$$\hat{y}_i^{(p)} = \boldsymbol{\alpha}^T \boldsymbol{X}_i + \beta_c^{(p)} c_i^{(p)},$$

where $c_i^{(p)}$ is the common variant PRS score of sample i for phenotype p and, as above, $\boldsymbol{X}_i$ is the vector of covariates for sample i. The weights learned during regression were $\boldsymbol{\alpha}$ (covariate weights) and $\beta_c^{(p)}$ (common variant PRS weight).

Extension with rare variants To incorporate the effects of rare variants into the phenotype predictors, we extended the common variant PRS models by the rare burden scores of significant genes, with models incorporating DeepRVAT or alternative burdens given respectively by

$$\hat{y}_i^{(p)} = \boldsymbol{\alpha}^T \boldsymbol{X}_i + \beta_c^{(p)} c_i^{(p)} + \sum_{j \in G_d^{(p)}} \beta_j^{(p)} \psi_r^*(V_{ij}),$$

$$\hat{y}_i^{(p)} = \boldsymbol{\alpha}^T \boldsymbol{X}_i + \beta_c^{(p)} c_i^{(p)} + \sum_{j \in G_b^{(p)}} \beta_j^{(p)} s_{ij}^p.$$

The difference lies in whether DeepRVAT or alternative burdens are used, and additionally the burdens and learned gene weights $\beta_j^{(p)}$ range over either the "Burden/SKAT combined" gene set $G_b^{(p)}$ or the DeepRVAT gene set $G_d^{(p)}$. The effect of gene set choice is further assessed in Supplementary Fig. 11.

Model fitting The linear and logistic regression models were fit in `R` v4.2.0 using the functions `lm` and `glm` (resp.) from the `stats` package using the family `binomial()` for logistic regression models, and otherwise retaining the default parameters.

Evaluation All models were fit on the training samples. The trained models were used to generate predictions on the evaluation samples. All evaluations were carried out on this set of predictions.

[4]Waist-to-Hip Ratio was excluded, since no PRS scores were available for this phenotype

1.6.5 Phenotype predictor assessment

Prediction accuracy To start, we assessed the performance of the phenotype predictors using two metrics: coefficient of determination (R^2) for the linear models and area under the precision-recall curve ($AUPRC$) for the logistic models. We compared the performance of two phenotype predictors using only the common variant PRS or using both the common PRS and rare variant burdens. Next, we calculated the relative improvement of the model that leverages rare variant burdens compared to the common PRS-only model as

$$\text{Relative}\Delta M = \frac{M_{rare} - M_{PRS_{only}}}{M_{PRS_{only}}},$$

where M denotes $AUPRC$ or R^2, respectively. We compared the relative improvement of the rare variant model using DeepRVAT gene impairment scores to predictors using alternative rare variant burdens by a one-sided, paired Wilcoxon test.

Additional evaluations The next evaluation focused on individuals with strong deviations between the rare variant and common variant PRS predictor. For each phenotype and individual, we calculated the absolute difference between the predicted phenotype values obtained from a linear model using either the common variant PRS alone or the the common variant PRS together with the rare variant burdens. Subsequently, we ranked individuals based on the magnitude of this difference. At each rank, we determined the count of individuals exhibiting outlier phenotypes, specifically those falling within the top or bottom 1% of the phenotypic distribution. Finally, we tested the enrichment of phenotype predictor outliers in individuals with extreme phenotypes. Across a range of z-score phenotype outlier cutoffs, we identified individuals above the phenotypic cutoff and determined the proportion of these individuals with a predicted phenotype value exceeding the 99% quantile. Enrichment scores were scaled relative to the baseline population (z-score = 0) and compared to the common PRS-only model.

Correlation with burden heritability For 15 phenotypes common to our study and [44], we correlated the coefficient of determination (R^2) obtained from our linear regression models with the aggregated burden heritability reported by [44].

1.7 Evaluation of feature importance

Despite their enormous success in improving predictive performance for various tasks, deep learning models remain challenging to interpret, and thus are commonly referred to as black-box models. In genomics, in-silico mutagenesis is one way of explaining the impact of the input perturbations on the model outcome through forward propagation. However such experiments are computationally expensive, thus encouraging the use of alternative approaches such as DeepLIFT [45] and GradCAM [46]. For a given input, these back-propagation based solutions compute the contribution of each feature by backpropagating through the network from the corresponding prediction. In order to explain the importance of the variant annotations used in DeepRVAT, we conducted separate analyses for quantitative and binary annotations to provide comparisons within each of these types.

1.7.1 Quantitative annotations

SHAP We employed SHAP (SHapley Additive exPlanations), a game-theory based method that assigns an importance score for each feature (in our case, annotation) based on the change in the expected model prediction for each corresponding model output [47]. In particular, we used SHAP DeepExplainer (v0.41.0), which integrates the DeepLIFT algorithm to approximate SHAP values, to compute importance scores for all variant annotations that were used to train DeepRVAT for phenotype prediction.

Subsampling For each repeat used in Section 1.5 and associated trained gene impairment module, the SHAP DeepExplainer was trained on 3,000 individuals from the training set. The importance of the variant annotations was explained on 1,000 individuals from the validation set. (The training and validation sets were the same as those used in Section 1.5.) Subsampling was necessary due to computational constraints.

Importance scores SHAP values produced by the explainer have the same shape as the input data, which is `individuals x genes x annotations x variants`. We aggregated the absolute SHAP values on individual, gene and variant levels to obtain an importance vector with the size of `annotations` by computing the average. Finally, the SHAP value vectors from each repeat were averaged to obtain aggregated importance scores.

Robustness of subsampling In order to illustrate the robustness of subsampling for feature importance explanations, we repeated the above procedure 15 times, each time randomly selecting different subsets of individuals from the training and validation sets. The annotations highlighted as important by the SHAP DeepExplainer were robust to the changes in the training and validation individuals. The final feature importance analyses were based on the aggregated SHAP values of the 15 different subsamplings.

1.7.2 Binary annotations

The impact of binary variant effect annotations was measured through in-silico mutagenesis experiments where only training (seed) genes were considered. For each binary annotation $\hat{l}$, we first created a filtered subset of individuals ($S_{\hat{l}}$) where each individual and gene pair has at least one annotated variant $v_k = (a_{kl})$ where $a_{k\hat{l}} = 1$ (wild type) in a given phenotype. A complementary mutant subset ($M_{\hat{l}}$) was created from $S_{\hat{l}}$ in which the corresponding annotation of the variants ($a_{k\hat{l}}$) were in-silico mutated to $a_{k\hat{l}} := 0$. The wild type ($S_{\hat{l}}$) and mutant ($M_{\hat{l}}$) individual subsets were separately scored using the DeepRVAT gene impairment module. The absolute difference between the gene impairment scores (aggregated over individuals and genes) was considered as the impact of the annotation of interest. Finally, a relative importance score for each annotation was calculated where the annotation with the highest absolute difference value was set to the relative importance value of 1. The relative importance of other annotations was scaled accordingly by dividing their difference scores by the maximum difference score value A, i.e., ($a_{\hat{l}}/A$).

1.7.3 Variant annotation groups

Groups Among the variant annotations that are utilized to train DeepRVAT, some have similar functionalities and are highly correlated (Supplementary Fig. 7). Therefore, in order to increase the interpretability of the feature importance analysis, we grouped certain annotations. Since UKBB MAF, CADD raw and DeepSEA principal components (PC1-PC6) were highlighted as relatively important compared to the rest of the continuous annotations by the SHAP explainer, we treated each of these annotations as a group in itself. The remaining annotations were assigned to a group (see (Supplementary Table 3).

DeepSEA We also assigned human-readable labels to first 6 DeepSEA principal components (PC1-PC6) that were used as annotations, based on the absolute values of the loadings (Supplementary Fig. 6) belonging to 919 epigenetic and regulatory genomic tracks. The principal components were named after the feature(s) within the top-ten highest loading values (Supplementary Fig. 6). For instance, we referred to DeepSEA PC2 as CTCF, since the five out of ten highest ranking loadings belonged to CTCF-related tracks.

1.8 Simulations

1.8.1 Genotype data

To compare power and type I error rates of DeepRVAT to conventional RVAT methods in simulation studies, we used semi-synthetic datasets generated based on real genotypes and annotations from the UK Biobank WES interim 200k release (more details in Sec. 1.5 below). To minimize confounding due to population structure, we restricted to 167,245 individuals of European genetic ancestry as determined by an analysis of genetic principal components [48].

1.8.2 Overview of phenotype simulation

Here, we give an overview of the phenotype simulation scheme. For the exact parameters see Supplementary Table 2.

Simulated causal genes We first sampled 100 genes and designated these as *simulated causal genes*. Gene sampling was restricted such that all sampled genes passed an expected allele frequency (EAF) filter of at least 50 for missense variants, and that 50% passed this filter for pLOF variants.

Variant impact score Next, variants in the simulated causal genes with MAF below the specified threshold were assigned an impact score based on selected variant annotations, namely variant MAF, 11 binary annotations (VEP consequences) and 5 continuous scores (SIFT, PolyPhen2, Condel, PrimateAI, CADD, AbSplice). (For more details on variant annotations, see Sec. 1.5.) Binary annotations were assigned a weight inversely proportional to their frequency, thereby reflecting the assumption that infrequent annotations, for example loss-of-function, have a larger impact. Continuous annotations were equally weighted. The variance explained by each variant score component (continuous annotations, binary annotations, MAF and variant score noise) is flexibly tunable.[5]

The primary aim of the simulation was to validate the model and specific properties, most importantly the ability of the model to learn ground truth effects and meaningful annotation filters from data, rather than requiring predefined cutoffs. To this end, it was not necessary to simulate realistic effect sizes per variant category.

Simulated causal variants, and gene burdens Based on the impact score (with noise added to introduce stochasticity), simulated causal variants were selected, such that a specified proportion of all variants was designated to be causal. Using these, gene burden scores were computed for each sample and each simulated causal gene by first aggregating variants individually for each annotation, followed by computing the weighted sum across the aggregated

[5] We note that since the annotations are not independent of one another, what we describe as the "variance explained" by each component cannot precisely be understood as such—for example, the sum of the "variance explained" by each component will be greater than the total variance explained by the annotations. Nevertheless, for simplicity, we will slightly abuse nomenclature by continuing to refer to this as "variance explained."

variant annotation scores. The genetic component of the simulated phenotype was then obtained as the sum of the gene burdens.

Simulated phenotypes Finally, to obtain the simulated phenotype values, we randomly sampled covariates and noise and rescaled each phenotype component (covariates, genetics, and noise) such that it explained the specified variance.

Allele frequency spectrum In Supplementary Fig. 4, we varied the effect size explained by variants with larger allele frequencies. To achieve this, we modified the weight assigned to binary annotations by employing weights defined as $q^{-1/x}$ for variant weighting, where q denotes the allele frequency (AF) and x takes on the values of 1, 3, and 10. As x increases, a greater number of variants with higher allele frequencies were selected as causal. The cumulative effect size explained by variants in each AF bin was determined as follows. Initially, we assigned a per-variant effect size to all simulated causal variants, which was computed by multiplying the simulated variant weight by the AF. Next, we calculated the cumulative effect size explained by each AF bin by summing the per-variant effect sizes within that particular bin and dividing this sum by the total summed effect sizes of variants from all AF bins. The cumulative effects explained by each AF bin were averaged across multiple simulation repeats.

1.8.3 Assessment of simulation results

Calibration To assess statistical calibration, phenotypic data was simulated from the null by sampling from a standard normal distribution, i.e., with no simulated causal genes or variants.

Power and type I error rates For all other simulation experiments, statistical power and type I error rates were assessed by comparison of discoveries (FWER < 0.05 as determined by the Benjamini-Hochberg procedure) to the simulated causal genes. For the rank-based evaluation (Supplementary Fig. 5b), we ranked gene-trait associations by their p-value as computed by DeepRVAT and the alternative methods. At each rank, we determined the cardinality of the intersection between the gene-trait associations at or below that rank and the set of simulated causal genes, and finally averaged the cardinality across simulation repeats.

1.9 Practical recommendations for users

1.9.1 Modes of usage

DeepRVAT can be applied in various modes, presented here in increasing levels of complexity. For each of these scenarios, we provide a corresponding Snakemake [49] pipeline.

Precomputed burden scores For users running association testing on UKBB WES data, we provide precomputed burden scores for all protein-coding genes with a qualifying variant within 300 bp of an exon, cf. Section 1.5.3. In this scenario, users are freed from processing of large WES data and may carry out highly computationally efficient association tests with the default DeepRVAT pipeline or the DeepRVAT+REGENIE integration.

Note that DeepRVAT scores are on a scale between 0 and 1, with a score closer to 0 indicating that the aggregate effect of variants in the gene is protective, and a score closer to 1 when the aggregate effect is deleterious.

Pretrained models Some users may wish to select variants or make variant-to-gene assigments differently from our methods, or to work on datasets other than UKBB. For this, we provide an ensemble of pretrained DeepRVAT gene impairment modules, which can be used for scoring individual-gene pairs for subsequent association testing. We also provide a pipeline for functional annotation of variants for compatibility with the pretrained modules.

Model training Other users may wish to exert full control over DeepRVAT scores, for example, to modify the model architecture, the set of annotations, or the set of training traits. For this, we provide pipelines for gene impairment module training, both in our CV and in a standard training/validation setup, with subsequent gene impairment score computation and association testing.

1.9.2 Gene impairment module training

For users wishing to train a custom DeepRVAT model, we provide here some practical suggestions based on our experiences (detailed in part in Extended Data Fig. 3).

Model architecture We found no benefit to using architectures larger than that used in this work, though we conjecture that larger architectures may provide some benefit with larger training data and more annotations. We performed limited experimentation with the aggregation function used (cf. 1.1.3) and found the maximum to give better results than the sum. However, exploring other choices or a learned aggregation remains open.

Training traits and seed genes We found that multiphenotype training improved performance, however, on our dataset, adding traits with fewer than three seed genes provided modest to no benefit. We also saw poor performance when including seed genes based on prior knowledge, e.g., known GWAS or RVAS associations, rather than the seed gene discovery methods (Section 1.1.4). We hypothesize that this is because an informative seed gene must have driver rare variants in the training dataset itself, which may not be the case for associations known from other cohorts.

Variant selection While association testing was carried out on variants with MAF $< 0.1\%$, we saw improved results when including a greater number of variants (we used MAF $< 1\%$) for training.

Variant annotations As seen in Extended Data Fig. 3, the best performance was achieved when including the full set of annotations, including correlated annotations. We thus recommend including annotations fairly liberally. However, we did find limits, for example, increasing the number of DeepSEA PCs from the 6 we used provided no benefit and eventually degraded model performance.

Model ensembling We found little to no benefit, but also no harm, from using more than 6 DeepRVAT gene impairment modules in our ensemble. Therefore, we chose this number as the most computationally efficient to achieve optimal results.

2 Supplementary Results

2.1 Model validation using simulated data

To validate DeepRVAT for gene discovery, we considered a semi-synthetic dataset derived from whole exome sequencing (WES) from 167,245 UK Biobank individuals of European ancestry (November 2020, 200k WES release [25]; **Methods**). We annotated all 12,704,497 WES variants using minor allele frequency (MAF), VEP [20] consequences, missense variant impact scores (SIFT [22] and PolyPhen2 [23], and omnibus statistical deleteriousness scores (CADD [50] and ConDel [51], as well as predicted annotations for effects on protein structure (PrimateAI[52], and aberrant splicing (AbSplice [30]. To create synthetic phenotypic data, we first simulated impairment scores for genes designated to be causally associated to the trait. To this end, we stochastically assigned each variant an effect based on its annotations (**Supplementary Fig. 2**), followed by aggregation for each gene. Finally, the individual phenotype values were simulated as a linear function of the gene impairment scores and additive effects from covariates mimicking age and sex, and independent Gaussian noise.

After confirming statistical calibration (Supplementary Fig. 3), we compared the power of DeepRVAT to conventional burden testing as well as variance component testing with SKAT [53], following Karczewski et al. [4], i.e., considering either predicted loss of function (pLOF) or missense variants and with weighting based on minor allele frequency (MAF). Notably, existing methods rely on a predefined MAF cutoff. To investigate the impact of a possible misspecification of the MAF cutoff, we varied the frequencies of the simulated causal variants (Supplementary Fig. 4, top to bottom). For each simulation setting, we applied the considered methods for alternative cutoff values of the MAF frequency (<0.01%, <0.1%, <1%; Supplementary Fig. 4, left to right). Whereas the power of conventional tests was greatly affected by the alignment of the simulated MAF distribution of causal variants and the MAF filter, DeepRVAT was consistently well powered, including in settings for which additional non-causal variants were incorporated in the MAF cutoff (Supplementary Fig. 4, bottom-right panel). Collectively, these results support that DeepRVAT is able to estimate an appropriate weighting for variants from the data, including implicit prioritization of variants by MAF.

Similarly, we considered altering the proportion of rare variants that are selected to contribute to gene impairment effects (Supplementary Fig. 1.3, Supplementary Fig. 5a) and a rank-based evaluation criterion (Supplementary Fig. 5b). Across these settings, DeepRVAT was consistently better powered than alternative methods. In sum, the benchmark using simulated data demonstrates that DeepRVAT yields results that are robust to a range of key parameters, including the MAF spectrum and the proportion of causal variants.

3 Supplementary Figures

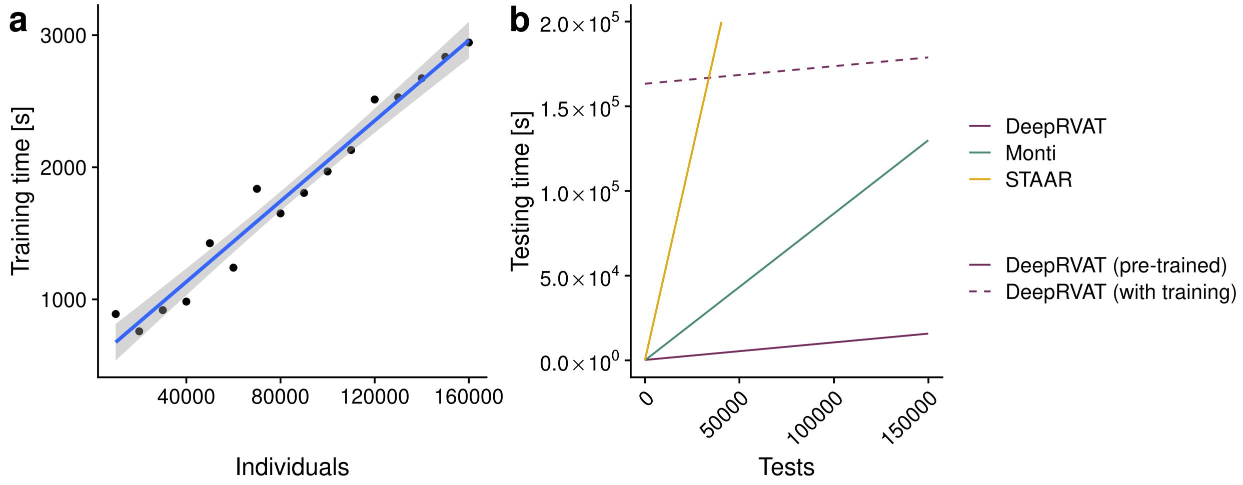

Supplementary Figure 1: Training and association testing times for DeepRVAT and comparison methods. (**a**) The UK Biobank WES was downsampled in intervals of 5,000 individuals and DeepRVAT was trained five times for each sample size. The plot indicates the mean training time in seconds, which scales roughly linearly with the sample size. Linear model fit with the 95% confidence interval shown in blue.
(b) Comparison of association testing times between DeepRVAT and alternative methods. Average compute time was determined by evaluating 1,000 tested genes for a single phenotype, resulting in the average time required to test one gene-phenotype pair. For STAAR and Monti et al.'s method, the total test time per gene was calculated by summing the compute times for individual variant filter masks or kernel designs and test types, respectively. The y-intercept of DeepRVAT (with training) represents the time needed for seed gene discovery and model training. The x-axis is truncated at 200,000 seconds. All timing experiments were conducted on a workstation with an AMD Ryzen Threadripper PRO 5975WX CPU and an NVIDIA RTX 4090 24GB GPU.

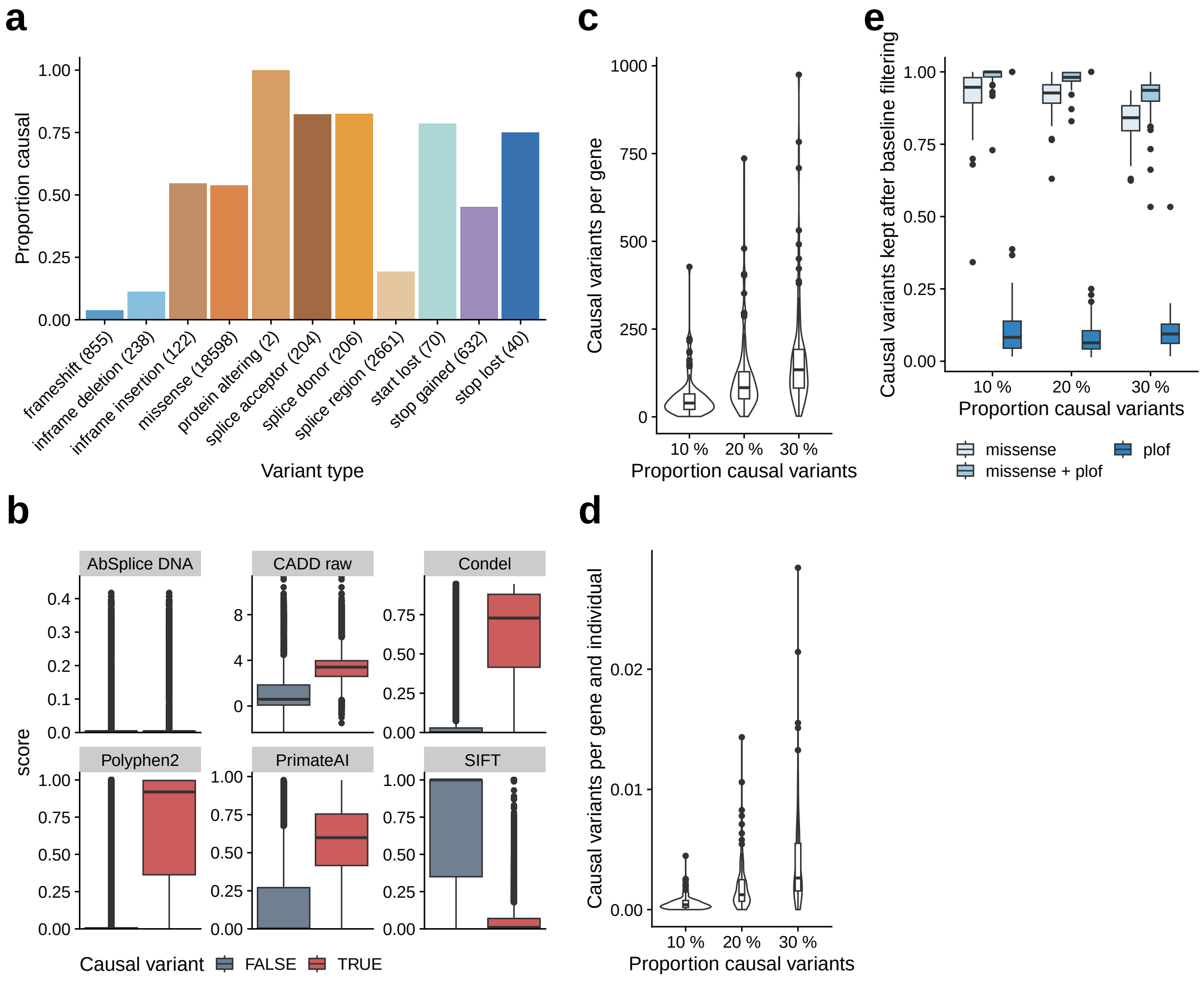

Supplementary Figure 2: Properties of simulated causal variants. Initially, a set of genes was selected as causal for the traits. To sample the causal variants, candidate variants were identified based on a selected variant AF threshold (**Supplementary Table 2**). These variants were then assigned an impact score calculated from the variant annotation profile, including variant AF, binary and continuous annotations and stochastic noise. Based on the impact score, simulated causal variants were selected such that a certain fraction of all candidate variants was designated to be causal. Box plots: Center line, median; box limits, first and third quartiles; whiskers span all data within 1.5 interquartile ranges of the lower and upper quartiles.
(**a - b**) Properties of simulated causal variants using default simulation parameters, as considered for Supplementary Fig. 3, Supplementary Fig. 4, and Supplementary Fig. 5b, while slightly varied parameters were employed for the remaining figures (**Supplementary Table 2**).
(**a**) The number of simulated causal variants relative to the total number of variants in a given binary annotation class (Average values across 10 simulation replicates). Numbers in parentheses indicate the total number of variants in a given category across all simulated causal genes.
(**b**) Distribution of annotation scores for continuous annotations, comparing simulated causal (n=10922) versus non-causal variants (n=61926). For all annotations but SIFT, higher scores indicate increased deleteriousness. The SIFT score decreases with deleteriousness.
(**c - e**) Variant statistics across different proportions of simulated causal variants, including the default value of 20%. Values are averaged across 10 simulation replicates.
(**c**) Number of causal variants for each gene designated to be causal. The observed variation reflects the simulation procedure as high-impact variants are selected irrespective of the gene they belong to.
(**d**) Number of causal variants per gene and individual, averaged across individuals for each gene.
(**e**) Proportion of simulated causal variants that are retained by the missense/pLOF variant filter as used in baseline methods. Across different proportions of causal variants, a high proportion of causal variants passes filtering for the baseline methods when combining missense/pLOF filter masks, which confirms that the baseline methods are, in theory, able to capture these simulated associations.

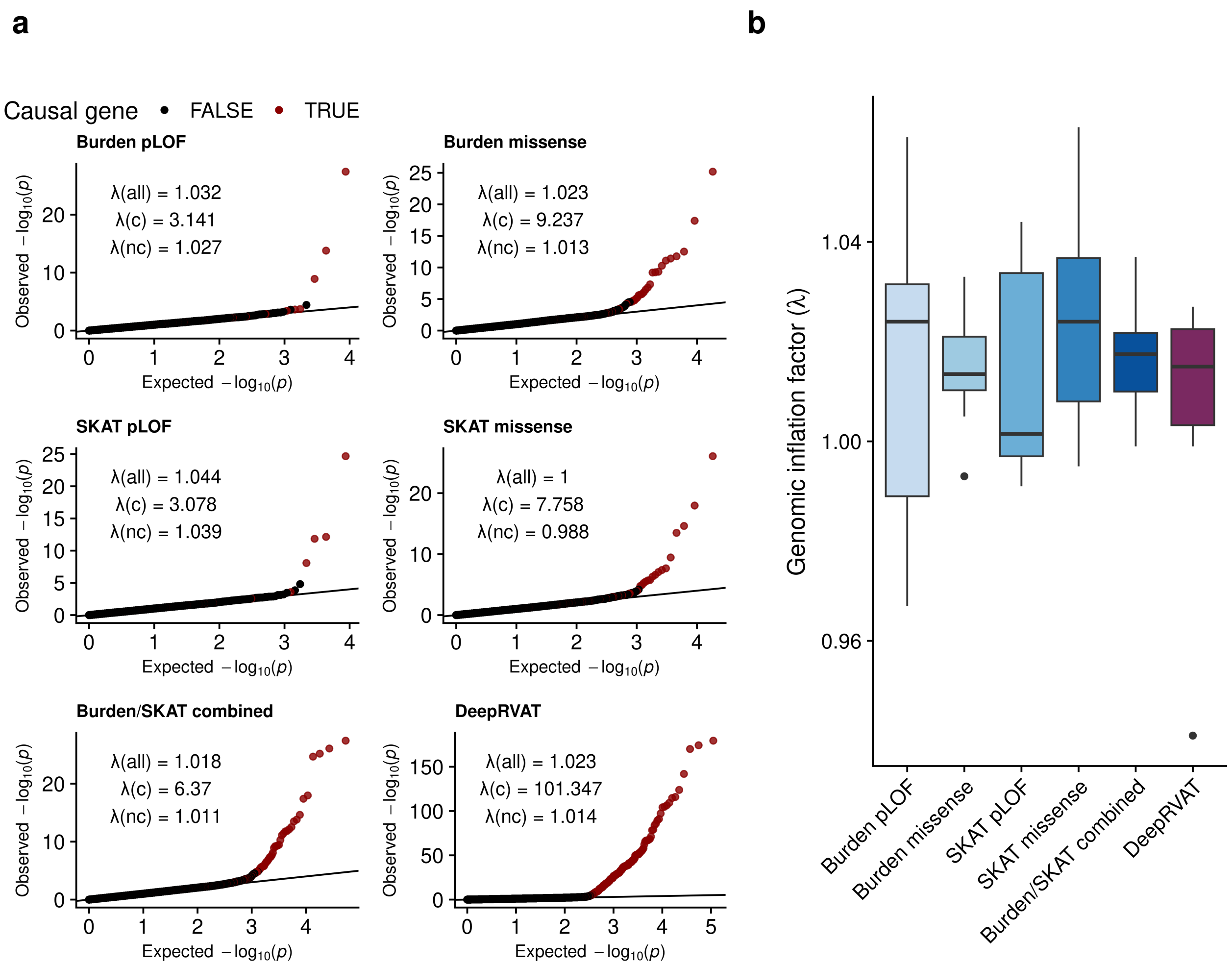

Supplementary Figure 3: Statistical calibration of DeepRVAT on simulated data. Considered were DeepRVAT using complete annotations, conventional burden tests and SKAT, each based on either pLOF or missense variants, and the combination of these four methods (Burden/SKAT combined).
(**a**) Assessment of calibration for DeepRVAT and alternative methods for an exemplary simulation replicate. Shown are Q-Q plots of the observed versus the expected association testing p-value distribution. The genomic inflation factor (λ) across all genes, either considering simulated causal (red), or non-causal genes (black) is reported.
(**b**) Genomic inflation factor λ_{GC}(genomic control) across 10 simulation replicates. Center line, median; box limits, first and third quartiles; whiskers span all data within 1.5 interquartile ranges of the lower and upper quartiles.

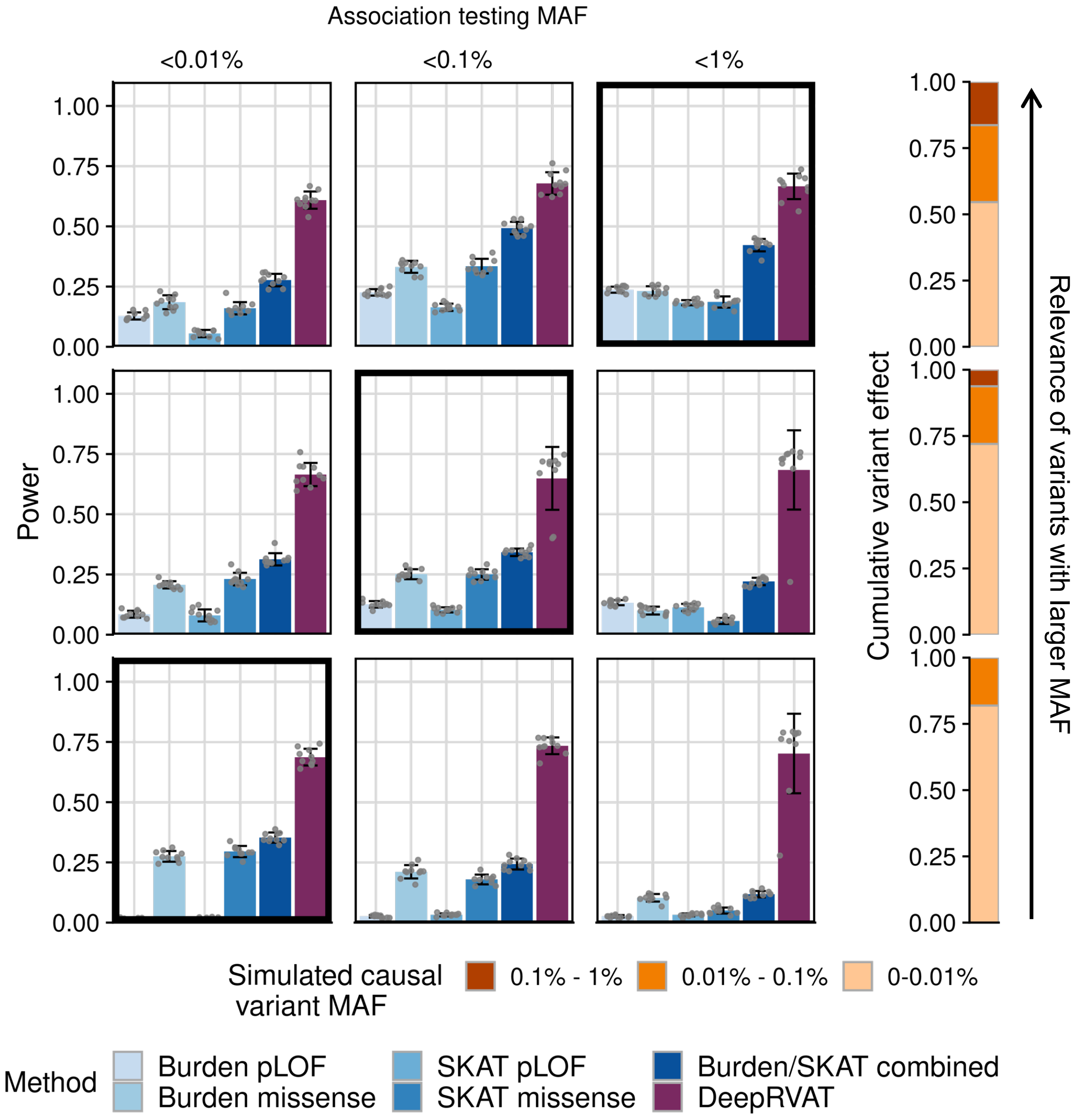

Supplementary Figure 4: Sensitivity of alternative methods to knowledge about the true relevance of different annotations. Power comparison for various MAF distributions of simulated causal variants (rows) versus MAF filters applied to determine input variants for alternative RVAT tests (columns). Shown is average power (bar height) and standard deviation (error bars) across 10 simulation runs (FWER $< 5\%$). Results on the diagonal correspond to the setting for which the MAF filter of the RVAT tests are aligned to the simulation setting; others correspond to varying degrees of MAF filter misspecification. Stacked bar plots on the right for each row denote the relative contribution of variants in different frequency bins for the respective simulation settings (averaged across all simulation runs). Simulation parameters for each row are detailed in **Supplementary Table 2**, with a consistent proportion of 20% simulated causal variants.

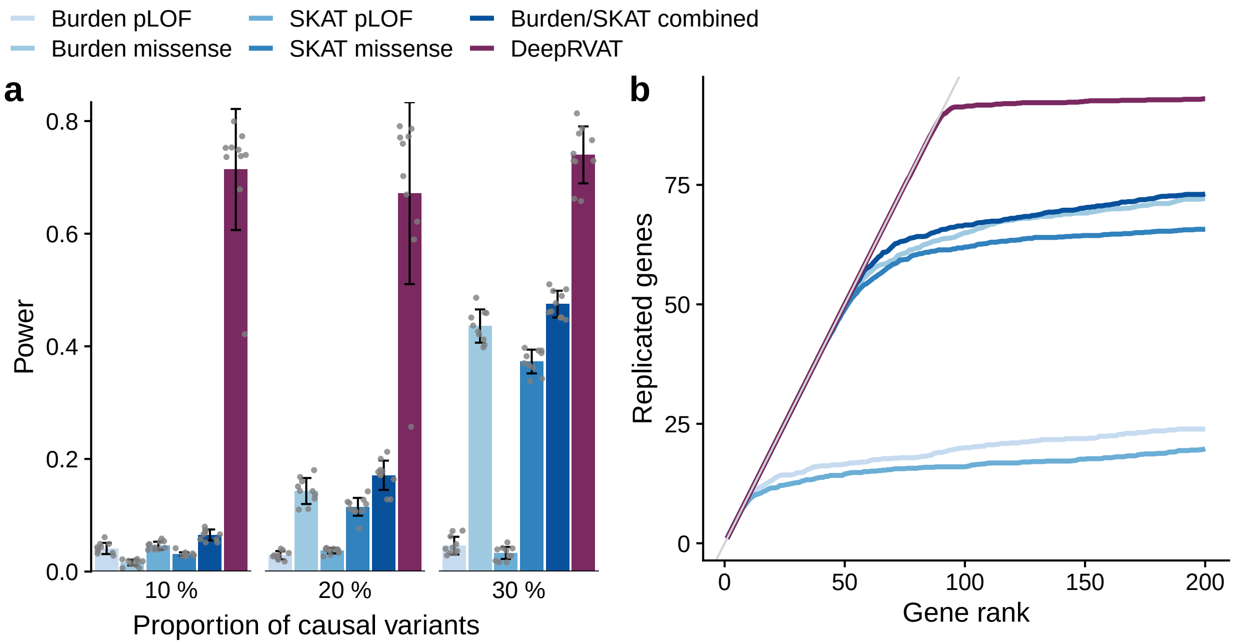

Supplementary Figure 5: Model assessment using simulated data. Comparison of DeepRVAT versus conventional burden or SKAT tests, using either pLOF or missense annotations, considering different simulation settings.
(**a**) Power of alternative methods for varying proportions of variants simulated to affect the trait. From left to right, 10%, 20%, and 30% of rare variants with MAF $< 0.1\%$ in the simulated causal genes were selected as causal variants. Bar height denotes average power across 10 simulated replicates (FWER$<$5%); error bars correspond to plus or minus one standard deviation.
(**b**) Rank-based evaluation. Genes were ranked by p-value and the proportion of simulated causal genes (true positives) at each rank was determined (average over 10 simulation replicates).

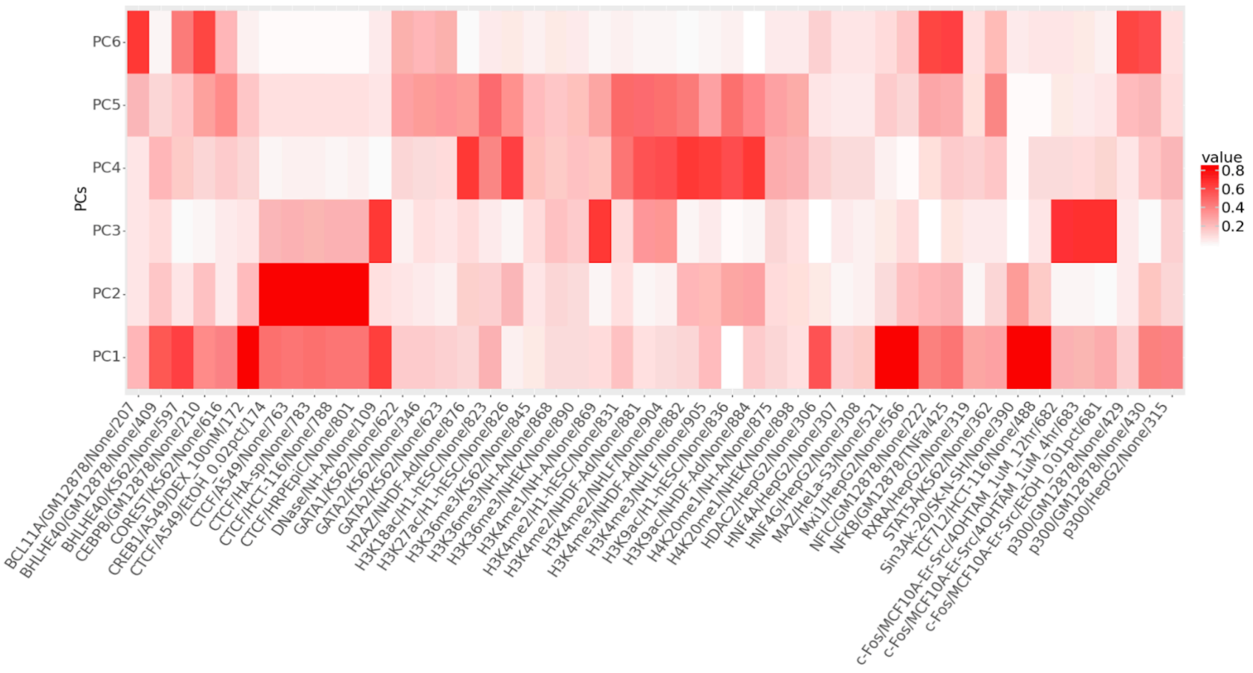

Supplementary Figure 6: Loadings for top six DeepSEA principal components. The top six DeepSEA principal components were used as annotations within DeepRVAT. Labels indicate feature name, cell type, treatment type, and position index among 919 original DeepSEA features.

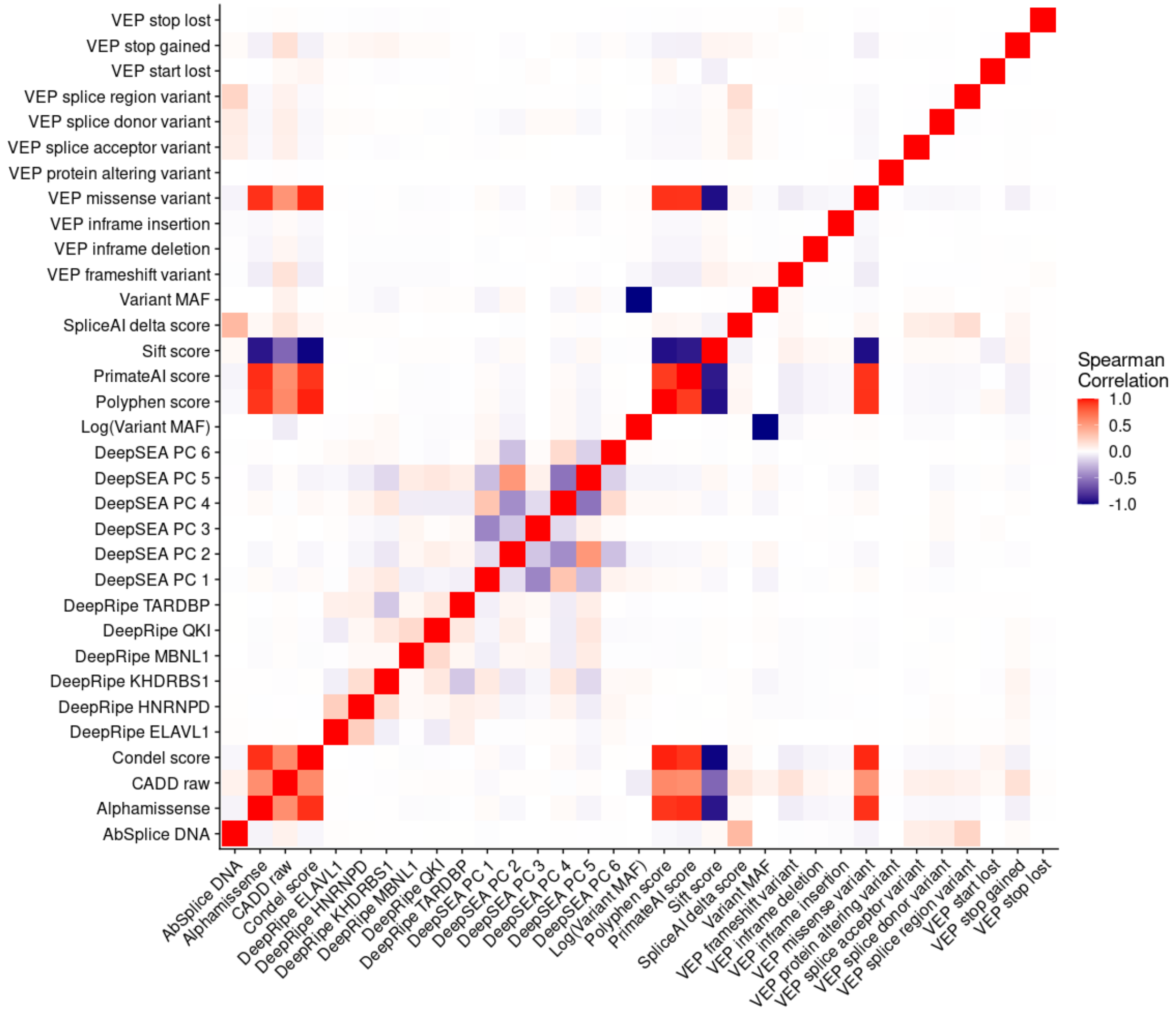

Supplementary Figure 7: Pairwise correlation of functional variant annotations. Heatmap showing Pearson correlation between 34 individual functional annotations using variants from the UKBB cohort. Unlike other scores, the SIFT score decreases with increasing deleteriousness, hence it is generally negatively correlated with other scores.

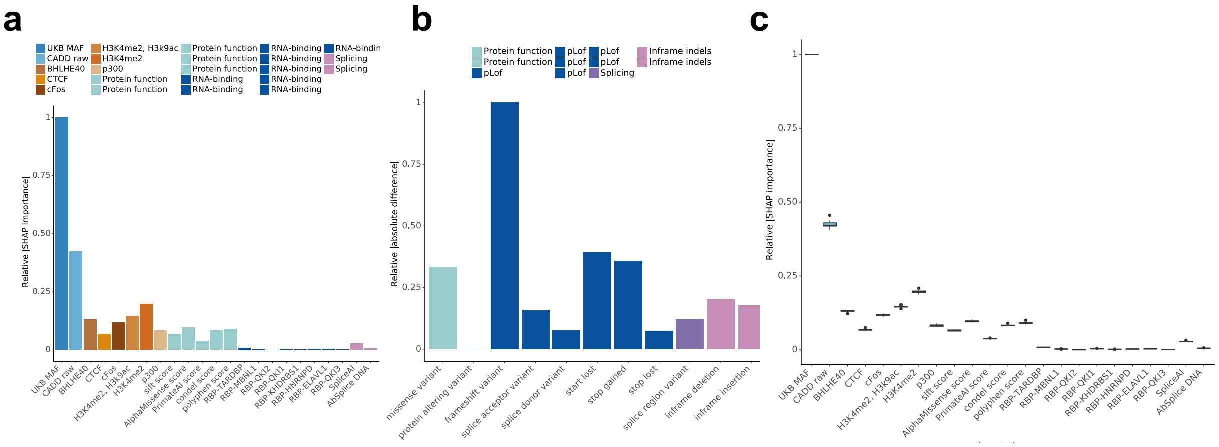

Supplementary Figure 8: Feature importance analysis. The relative importance scores displayed in plots range between 0-1 and are calculated such that the relative importance of the binary/quantitative annotation with the strongest impact is 1. The annotations are depicted with the same color if they belong to the same annotation group.

(a) *In-silico* mutagenesis analysis for binary annotations considered by DeepRVAT. The impact of a given binary annotation was assessed by comparing DeepRVAT predictions using the real annotation vector with a mutated annotation vector, where the effect of the considered binary annotation was masked (set to zero). The absolute difference between the two gene impairment scores, aggregated across all samples and genes, was considered as the impact of the variant of interest.

(b) SHAP importance values for quantitative annotations considered by DeepRVAT. In order to assess the contribution of quantitative annotations to the DeepRVAT predictions, we utilized SHAP DeepExplainer. The explainer was trained on 3,000 samples from the training set and 1,000 samples from the validation set were used to obtain SHAP importance values from the trained explainer. The output of the explainer had the same shape as the input. Therefore, the absolute SHAP values were aggregated to obtain annotation-level importance scores. This procedure was repeated 15 times with different training and validation samples. The final scores were computed by the aggregation over 15 different samplings.

(c) The relative SHAP importance scores for quantitative annotations are robust across 15 different samplings. The relative SHAP importance scores of quantitative annotations are plotted across 15 samplings, where different training and validation samples were fed to the explainer model. Center line, median; box limits, first and third quartiles; whiskers span all data within 1.5 interquartile ranges of the lower and upper quartiles.

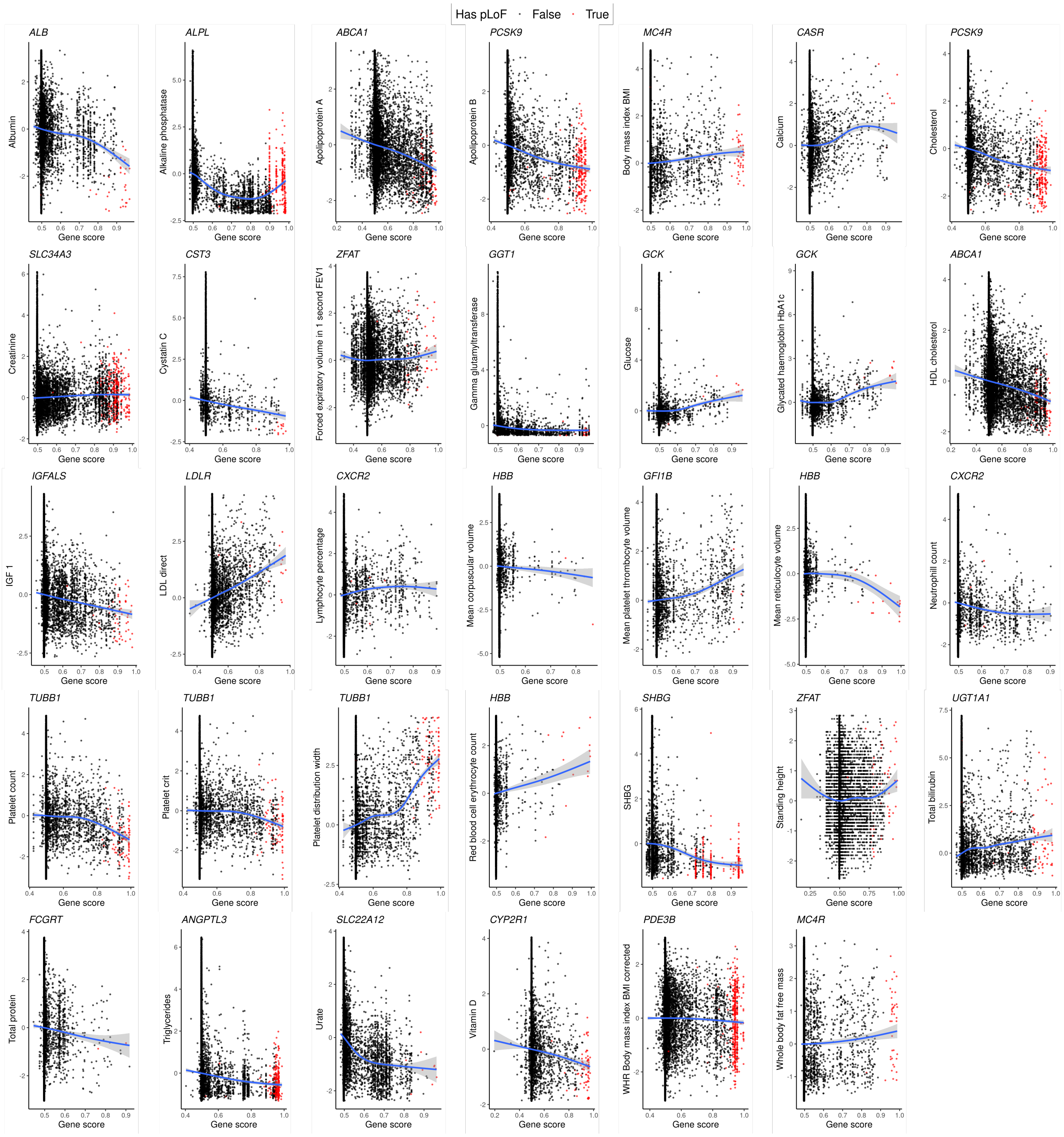

Supplementary Figure 9: DeepRVAT gene impairment score versus trait measurement. Scatterplot of DeepRVAT gene impairment scores vs. trait measurements for selected, associated genes for all 34 quantitative traits. Each point represents one individual from the cohort. Red points indicate that the corresponding individual has at least one pLOF variant in the gene. Blue line denotes the GAM fit with shaded areas corresponding to 95% confidence intervals.

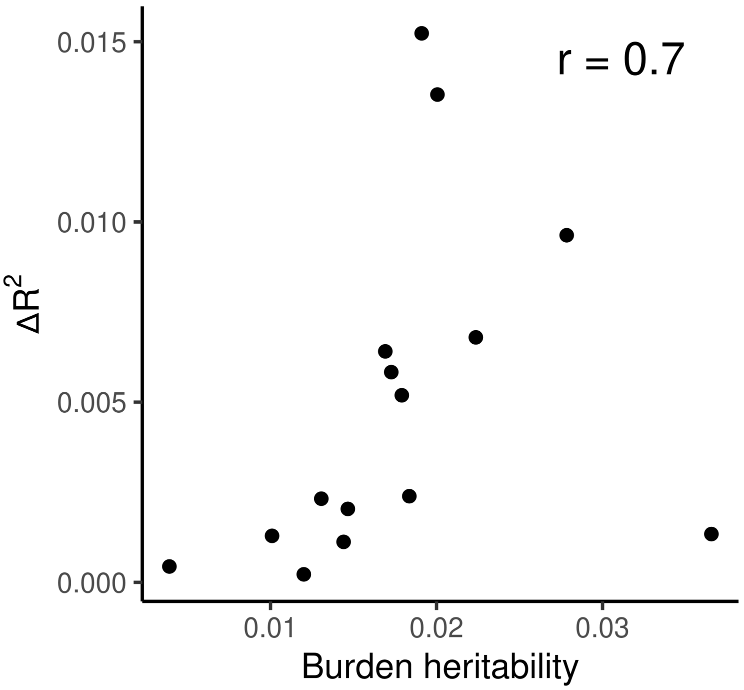

Supplementary Figure 10: Correlation of Delta R2 of rare variant phenotype predictors with burden heritability. For 15 phenotypes common to both, our study and Weiner, Nadig et al.'s work[44], we correlated the difference in the coefficient of determination (R^2) for models that account for rare variants versus a common-variant PRS model with the aggregated burden heritability. Burden heritability quantifies the proportion of phenotypic variance explained by the gene-wise burden of rare variants[44]. The Spearman correlation coefficient is reported.

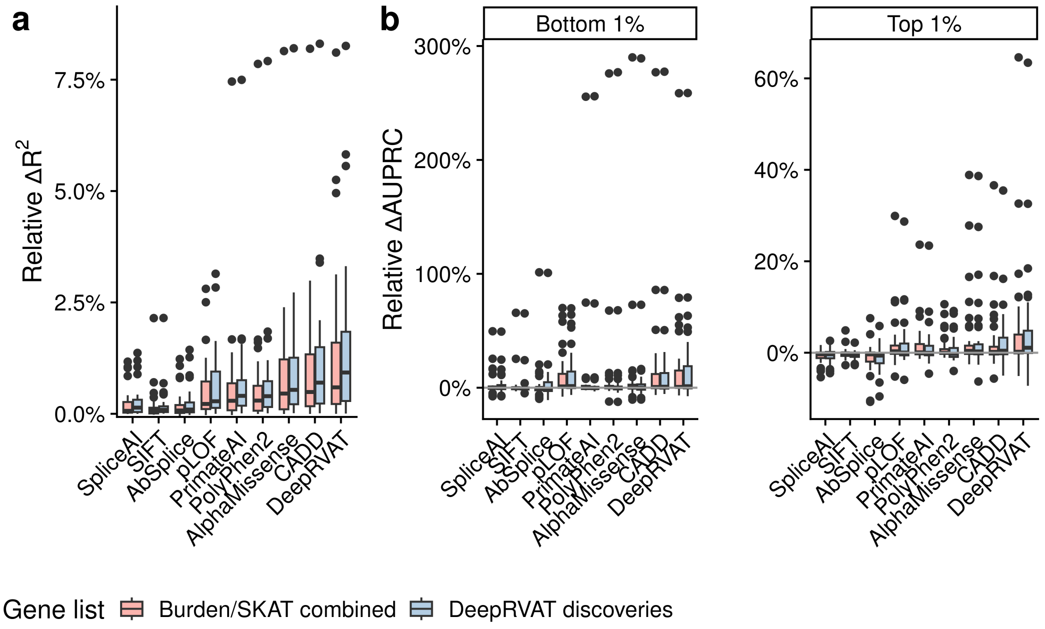

Supplementary Figure 11: Impact of gene list choice on the performance of rare variant phenotype predictors. For each rare variant gene burden, we compared the performance of rare variant phenotype predictors including gene burdens from genes identified through conventional RVAT methods (Burden/SKAT combined) or DeepRVAT (FWER $< 5\%$) across 33 UK Biobank traits. Center line, median; box limits, first and third quartiles; whiskers span all data within 1.5 interquartile ranges of the lower and upper quartiles.

(**a**) Relative improvement of the prediction performance of a linear regression model that includes rare variant gene burdens versus a model based on common variant PRS only. DeepRVAT gene impairment scores consistently outperform alternative rare variant phenotype predictors, regardless of the gene list choice. Additionally, incorporating genes discovered by DeepRVAT further enhances the performance compared to using genes identified by conventional RVAT methods.

(**b**) Analogous comparison as in **a**, however considering a logistic regression model to stratify individuals in the bottom or top 1% of the phenotypic distribution. Shown are relative differences in the area under the precision-recall curve (AUPRC) between a model that includes rare variant gene burdens versus a PRS-only model.

4 Supplementary Table Captions

Supplementary Table 1. Conceptual comparison of DeepRVAT to related methods.

Supplementary Table 2. Simulation parameters. For each figure related to the simulation studies, simulation parameters used with the phenotype simulation algorithm as described in **Methods** are listed.

Supplementary Table 3. Variant annotations used within DeepRVAT. The table presents a comprehensive description of each variant annotation utilized, including the source of the annotation score, the assigned group in the feature importance analysis (Supplementary Fig. 9), and whether the annotation was employed by the method proposed by Monti et al.

Supplementary Table 4. Quantitative and clinical phenotypes from UK Biobank used in this study. *Columns:* Trait: Name of analyzed trait; Trait description: More detailed trait name; UKBB Data Field: UK Biobank data field ID corresponding to trait; samples_*: Sample sizes or case/control counts for both the cohort used in Figure 2 (161k, Table 6) and for the complete all ancestry UK Biobank dataset (470k_aa, Supplementary Table 9); PRS ID: PGS catalog id (`https://www.pgscatalog.org/`) from which variants and effect sizes for the common variant PRS calculation were retrieved; Used for DeepRVAT training: Indicates if the phenotype was used for training the DeepRVAT gene impairment module; Trait Group: Trait group the trait was assigned to for plots in Figure 2 and Figure 4.

Supplementary Table 5. Covariates used as controls association testing as well as DeepRVAT training. *Columns:* Covariate: Name of covariate; UKB field ID: UK Biobank field ID for covariate.

Supplementary Table 6. Significant gene-trait associations discovered by DeepRVAT in 161,822 unrelated individuals of European ancestry from the UK Biobank. The unadjusted association testing *p*-value as returned by the SEAK score test function from the unconditional and conditional analysis is reported for each significant gene-trait combination. Discoveries previously reported in UKBB WES studies on the full UKBB cohort(Backman et al., 2021 [5]; Karczewski et al., 2022 [4]; Supplementary Table 7) are indicated in the 'Known from previous UKBB WES studies' column.

Supplementary Table 7. Gene-trait associations discovered in other UK Biobank association studies on larger cohorts. The significant associations, as defined by Backman et al. 2021 [5] or Karczewski et al. 2022 (Genebass) [4], are listed for all traits examined in this paper.

Supplementary Table 8. Phenotype prediction results. Columns: Trait: Name of analyzed trait; Gene list: Genes included in the phenotype predictor. Either discoveries from Burden/SKAT combined or DeepRVAT; Rare burden type: Rare variant burden type; Metric: Phenotype predictor performance metric; Metric PRS: performance with covariates and common variant PRS; Metric rare burden: performance with rare variant gene burdens in addition to common variant PRS and covariates ; Delta Metric: improvement in performance from including rare gene burdens (Metric rare burden - Metric PRS); Relative Delta Metric: Delta Metric/ Metric PRS.

Supplementary Table 9. Significant gene trait associations discovered by DeepRVAT + REGENIE across 34 quantitative and 63 disease traits in the entire UKBB cohort of 469,382 individuals with available WES. For each significant gene-trait pair, unadjusted p-values and betas as returned by REGENIE from both unconditional and conditional analyses are provided. Results are also reported for the subset of individuals of European ancestry (N=409,519). Discoveries previously reported in other UKBB WES studies (Backman et al., 2021 [5]; Karczewski et al., 2022 [4], Jurgens et al., 2022 [36]) are indicated in the 'Known from previous UKBB WES studies' column.

References

[1] Manzil Zaheer et al. "Deep Sets". *Advances in Neural Information Processing Systems* 30 (2017).

[2] Ilya Loshchilov and Frank Hutter. "Decoupled weight decay regularization". *arXiv preprint arXiv:1711.05101* (2017).

[3] Ole Tange. *Gnu Parallel 2018*. Apr. 27, 2018.

[4] Konrad J. Karczewski et al. "Systematic single-variant and gene-based association testing of thousands of phenotypes in 394,841 UK Biobank exomes". *Cell Genomics* 2.9 (Sept. 2022).

[5] Joshua D. Backman et al. "Exome sequencing and analysis of 454,787 UK Biobank participants". *Nature* 599.7886 (Nov. 25, 2021).

[6] Bo Eskerod Madsen and Sharon R. Browning. "A Groupwise Association Test for Rare Mutations Using a Weighted Sum Statistic". *PLoS Genetics* 5.2 (Feb. 13, 2009). Ed. by Nicholas J. Schork.

[7] Iuliana Ionita-Laza et al. "Sequence kernel association tests for the combined effect of rare and common variants". *American Journal of Human Genetics* 92.6 (June 6, 2013).

[8] Seunggeun Lee et al. "Optimal Unified Approach for Rare-Variant Association Testing with Application to Small-Sample Case-Control Whole-Exome Sequencing Studies". *The American Journal of Human Genetics* 91.2 (Aug. 2012).

[9] Gundula Povysil et al. "Rare-variant collapsing analyses for complex traits: guidelines and applications". en. *Nature Reviews Genetics* 20.12 (2019).

[10] Seunggeung Lee et al. "Rare-Variant Association Analysis: Study Designs and Statistical Tests". *The American Journal of Human Genetics* 95.1 (July 2014).

[11] Remo Monti et al. "Identifying interpretable gene-biomarker associations with functionally informed kernel-based tests in 190,000 exomes". *Nature Communications* 13.1 (Sept. 10, 2022).

[12] Jianping Sun, Yingye Zheng, and Li Hsu. "A Unified Mixed-Effects Model for Rare-Variant Association in Sequencing Studies". *Genetic Epidemiology* 37.4 (May 2013).

[13] Hana Susak et al. "Efficient and flexible Integration of variant characteristics in rare variant association studies using integrated nested Laplace approximation". *PLOS Computational Biology* 17.2 (Feb. 19, 2021). Ed. by Yue Li.

[14] Zihuai He et al. "Unified Sequence-Based Association Tests Allowing for Multiple Functional Annotations and Meta-analysis of Noncoding Variation in Metabochip Data". *The American Journal of Human Genetics* 101.3 (Sept. 2017).

[15] Xihao Li et al. "Dynamic incorporation of multiple in silico functional annotations empowers rare variant association analysis of large whole-genome sequencing studies at scale". *Nature Genetics* 52.9 (Sept. 2020).

[16] Stefan Konigorski, Shahryar Khorasani, and Christoph Lippert. "Integrating omics and MRI data with kernel-based tests and CNNs to identify rare genetic markers for Alzheimer's disease". *arXiv:1812.00448 [cs, q-bio, stat]* (Mar. 5, 2019). arXiv: `1812.00448`.

[17] Yaowu Liu and Jun Xie. "Cauchy Combination Test: A Powerful Test With Analytic p -Value Calculation Under Arbitrary Dependency Structures". *Journal of the American Statistical Association* 115.529 (Jan. 2, 2020).

[18] Yaowu Liu et al. "ACAT: A Fast and Powerful p Value Combination Method for Rare-Variant Analysis in Sequencing Studies". *The American Journal of Human Genetics* 104.3 (Mar. 2019).

[19] Joelle Mbatchou et al. "Computationally efficient whole-genome regression for quantitative and binary traits". *Nature Genetics* 53.7 (July 2021).

[20] William McLaren et al. "The Ensembl Variant Effect Predictor". *Genome Biology* 17.1 (Dec. 2016).

[21] Wei Zhou et al. "SAIGE-GENE+ improves the efficiency and accuracy of set-based rare variant association tests". *Nature genetics* 54.10 (2022).

[22] Prateek Kumar, Steven Henikoff, and Pauline C Ng. "Predicting the effects of coding non-synonymous variants on protein function using the SIFT algorithm". *Nature Protocols* 4.7 (July 2009).

[23] Ivan A Adzhubei et al. "A method and server for predicting damaging missense mutations". *Nature Methods* 7.4 (Apr. 2010).

[24] Gavin Band and Jonathan Marchini. *BGEN: a binary file format for imputed genotype and haplotype data*. Apr. 29, 2018.

[25] Joseph D. Szustakowski et al. "Advancing human genetics research and drug discovery through exome sequencing of the UK Biobank". *Nature Genetics* 53.7 (July 2021).

[26] Cristopher V. Van Hout et al. "Exome sequencing and characterization of 49,960 individuals in the UK Biobank". *Nature* 586.7831 (Oct. 29, 2020).

[27] Petr Danecek et al. "Twelve years of SAMtools and BCFtools". *GigaScience* 10.2 (Jan. 29, 2021).

[28] Jian Zhou and Olga G. Troyanskaya. "Predicting effects of noncoding variants with deep learning–based sequence model". *Nature Methods* 12.10 (Oct. 2015).

[29] Kishore Jaganathan et al. "Predicting Splicing from Primary Sequence with Deep Learning". *Cell* 176.3 (Jan. 24, 2019).

[30] Nils Wagner et al. "Aberrant splicing prediction across human tissues". *Nature Genetics* 55.5 (2023).

[31] Mahsa Ghanbari and Uwe Ohler. "Deep neural networks for interpreting RNA-binding protein target preferences". *Genome research* 30.2 (2020).

[32] *DeepRipe*. `https://github.com/ohlerlab/DeepRiPe`. 2022 (Online; accessed July, 2022).

[33] Gina M Peloso et al. "Association of low-frequency and rare coding-sequence variants with blood lipids and coronary heart disease in 56,000 whites and blacks". *The American Journal of Human Genetics* 94.2 (2014).

[34] Margaret Sunitha Selvaraj et al. "Whole genome sequence analysis of blood lipid levels in¿ 66,000 individuals". *Nature communications* 13.1 (2022).

[35] Yann C Klimentidis et al. "Phenotypic and genetic characterization of lower LDL cholesterol and increased type 2 diabetes risk in the UK Biobank". *Diabetes* 69.10 (2020).

[36] Sean J. Jurgens et al. "Analysis of rare genetic variation underlying cardiometabolic diseases and traits among 200,000 individuals in the UK Biobank". *Nature Genetics* (Feb. 17, 2022).

[37] Ken B Hanscombe et al. "ukbtools: An R package to manage and query UK Biobank data". *PLoS One* 14.5 (2019).

[38] the Haplotype Reference Consortium. "A reference panel of 64,976 haplotypes for genotype imputation". *Nature Genetics* 48.10 (Oct. 2016).

[39] Jie Huang et al. "Improved imputation of low-frequency and rare variants using the UK10K haplotype reference panel". *Nature Communications* 6.1 (Sept. 14, 2015).

[40] Christopher C Chang et al. "Second-generation PLINK: rising to the challenge of larger and richer datasets". *GigaScience* 4.1 (Dec. 2015).

[41] Konrad J Karczewski et al. "Pan-UK Biobank GWAS improves discovery, analysis of genetic architecture, and resolution into ancestry-enriched effects". *medRxiv* (2024).

[42] Samuel A. Lambert et al. "The Polygenic Score Catalog as an open database for reproducibility and systematic evaluation". en. *Nature Genetics* 53.44 (2021).

[43] Florian Privé et al. "Portability of 245 polygenic scores when derived from the UK Biobank and applied to 9 ancestry groups from the same cohort". en. *The American Journal of Human Genetics* 109.1 (2022).

[44] Daniel J. Weiner et al. "Polygenic architecture of rare coding variation across 394,783 exomes". *Nature* 614.7948 (Feb. 16, 2023).

[45] Avanti Shrikumar, Peyton Greenside, and Anshul Kundaje. “Learning important features through propagating activation differences”. In: *International conference on machine learning*. PMLR. 2017.

[46] Ramprasaath R Selvaraju et al. “Grad-cam: Visual explanations from deep networks via gradient-based localization”. In: *Proceedings of the IEEE international conference on computer vision*. 2017.

[47] Scott M Lundberg and Su-In Lee. “A unified approach to interpreting model predictions”. *Advances in neural information processing systems* 30 (2017).

[48] Clare Bycroft et al. “The UK Biobank resource with deep phenotyping and genomic data”. *Nature* 562.7726 (Oct. 2018).

[49] Felix Mölder et al. “Sustainable data analysis with Snakemake”. *F1000Research* 10 (Apr. 19, 2021).

[50] Philipp Rentzsch et al. “CADD: predicting the deleteriousness of variants throughout the human genome”. *Nucleic Acids Research* 47 (D1 Jan. 8, 2019).

[51] Abel González-Pérez and Nuria López-Bigas. “Improving the Assessment of the Outcome of Nonsynonymous SNVs with a Consensus Deleteriousness Score, Condel”. *The American Journal of Human Genetics* 88.4 (Apr. 2011).

[52] Laksshman Sundaram et al. “Predicting the clinical impact of human mutation with deep neural networks”. *Nature Genetics* 50.8 (Aug. 2018).

[53] Michael C. Wu et al. “Rare-Variant Association Testing for Sequencing Data with the Sequence Kernel Association Test”. *The American Journal of Human Genetics* 89.1 (July 2011).
