# Supplementary material for: Integration of variant annotations using deep set networks boosts rare variant association testing
Source: Nat Genet. 2024 Sep 25;56(10):2271–80. doi: 10.1038/s41588-024-01919-z (PMC11525182; doi:10.1038/s41588-024-01919-z)
Supplement: Supplementary file 2 — Reporting Summary [file 41588_2024_1919_MOESM2_ESM.pdf]

nature portfolio

Corresponding author(s): Brian Clarke, Oliver Stegle, Julien Gagneur

Last updated by author(s): 2024/07/24

Reporting Summary

Nature Portfolio wishes to improve the reproducibility of the work that we publish. This form provides structure for consistency and transparency in reporting. For further information on Nature Portfolio policies, see our Editorial Policies and the Editorial Policy Checklist.

Statistics

For all statistical analyses, confirm that the following items are present in the figure legend, table legend, main text, or Methods section.

n/a	Confirmed	
[ ]	[x]	The exact sample size (*n*) for each experimental group/condition, given as a discrete number and unit of measurement
[ ]	[x]	A statement on whether measurements were taken from distinct samples or whether the same sample was measured repeatedly
[ ]	[x]	The statistical test(s) used AND whether they are one- or two-sided<br>*Only common tests should be described solely by name; describe more complex techniques in the Methods section.*
[ ]	[x]	A description of all covariates tested
[ ]	[x]	A description of any assumptions or corrections, such as tests of normality and adjustment for multiple comparisons
[ ]	[x]	A full description of the statistical parameters including central tendency (e.g. means) or other basic estimates (e.g. regression coefficient) AND variation (e.g. standard deviation) or associated estimates of uncertainty (e.g. confidence intervals)
[ ]	[x]	For null hypothesis testing, the test statistic (e.g. *F*, *t*, *r*) with confidence intervals, effect sizes, degrees of freedom and *P* value noted<br>*Give P values as exact values whenever suitable.*
[x]	[ ]	For Bayesian analysis, information on the choice of priors and Markov chain Monte Carlo settings
[x]	[ ]	For hierarchical and complex designs, identification of the appropriate level for tests and full reporting of outcomes
[ ]	[x]	Estimates of effect sizes (e.g. Cohen's *d*, Pearson's *r*), indicating how they were calculated

Our web collection on statistics for biologists contains articles on many of the points above.

Software and code

Policy information about availability of computer code

Data collection	No data collection was performed. No software was used for data collection.
Data analysis	DeepRVAT(v1.0) was used in both simulation and real data analysis and is implemented as an open-source python package available at https://github.com/PMBio/deeprvat/.<br>Pre-trained DeepRVAT models are available on Zenodo: https://doi.org/10.5281/zenodo.12772611.<br>The code for downstream analyses is available at https://github.com/PMBio/deeprvat-analysis/.<br>For association testing, the SEAK package (0.4.3) was used https://seak.readthedocs.io/en/latest/.<br>Data analysis was performed using python (3.8) and R (4.2). All modules used for DeepRVAT training and data postprocessing are listed in https://github.com/PMBio/deeprvat/blob/main/deeprvat_env.yaml. Modules required for analyses in R are provided here https://github.com/PMBio/deeprvat-analysis/blob/main/r-env.yaml.<br>UK Biobank data preprocessing was done using our custom preprocessing pipeline available at https://github.com/PMBio/deeprvat/blob/main/deeprvat/preprocessing/README.md using bcftools (1.10.2) and samtools (1.9).<br>The bgen package (v1.6.1) was used to convert the (samples genes) matrix of DeepRVAT scores to a BGEN file of pseudovariants.<br>Association testing using REGENIE was done using the REGENIE package (v3.4) https://github.com/rgcgithub/regenie/<br>Independently associated variants were identified from GWAS summary statistics through LD-based clumping using PLINK (v1.9).<br>DeepSEA predictions were obtained using Kipoi-veff2 (https://github.com/kipoi/kipoi-veff2).<br>DeepRIPE predictions were obtained using the code provided here https://github.com/ohlerlab/DeepRiPe.<br>All remaining variant annotations (excluding AbSplice, SpliceAI and AlphaMissense) were obtained using VEP v109.

For manuscripts utilizing custom algorithms or software that are central to the research but not yet described in published literature, software must be made available to editors and reviewers. We strongly encourage code deposition in a community repository (e.g. GitHub). See the Nature Portfolio guidelines for submitting code & software for further information.

Data

Policy information about availability of data

All manuscripts must include a data availability statement. This statement should provide the following information, where applicable:

- Accession codes, unique identifiers, or web links for publicly available datasets
- A description of any restrictions on data availability
- For clinical datasets or third party data, please ensure that the statement adheres to our policy

AlphaMissense scores were obtained from https://www.google.com/url?q=https://storage.googleapis.com/dm_alphamissense/AlphaMissense_hg38.tsv.gz&sa=D&source=editors&ust=1714037736792761&usg=AOvVaw34CM435oT9SM5ziM6SQn2-.
AbSplice and SpliceAI scores were obtained from Zenodo (https://zenodo.org/record/6631476).
PRS Scores were obtained from https://www.pgscatalog.org/ using PRS ids provided in Supplementary Table 3.
GWAS summary statistics were obtained from the Pan-UK Biobank resource (https://pan.ukbb.broadinstitute.org , Karczewski et al., medRxiv 2024).
The UK Biobank analyses were conducted using the UK Biobank resource (Project IDs 25214, 44108, and 81358).
Replication data was retrieved from genebass gs://ukbb-exome-public/500k/results/results.mt and and the study by Backman et al., 2021 (https://doi.org/10.1038/s41586-021-04103-z) (https://static-content.springer.com/esm/art%3A10.1038%2Fs41586-021-04103-z/MediaObjects/41586_2021_4103_MOESM5_ESM.xlsx SD2) and is also provided in Supplementary Table 7.
GENCODE release 38 can be downloaded from https://www.gencodegenes.org/human/release_38.html
The association testing results from DeepRVAT+REGENIE on the 500k UK Biobank dataset, covering all genes and traits for all ancestries and Caucasians only, are available on Zenodo https://doi.org/10.5281/zenodo.12736824.

Research involving human participants, their data, or biological material

Policy information about studies with human participants or human data. See also policy information about sex, gender (identity/presentation), and sexual orientation and race, ethnicity and racism.

Reporting on sex and gender	The genetic sex of participants was accessed through UK-Biobank Data-Field 22001, and included as a covariate in the statistical analyses. The genetic sex was determined by a genotyping analysis as described here: https://biobank.ndph.ox.ac.uk/showcase/field.cgi?id=22001<br><br>Of the 469,835 individuals considered in this study, 254,489 were estimated to be female and 214,893 estimated to be male by the genotyping analysis. We note that the estimated genetic sex can differ from the self-reported sex from UK Biobank Data Field 31.
Reporting on race, ethnicity, or other socially relevant groupings	To minimize confounding due to population structure, we restricted to 161,822 unrelated individuals of Caucasian genetic ethnicity as determined by an analysis of genetic principal components for our benchmarking analyses. For biological discovery, we used individuals of all ancestry from the UK Biobank.
Population characteristics	The mean age at recruitment of the 469,835 participants included in the statistical analyses in this study was 56.54, standard deviation 8.10, and ranged from 37 to 73 years. The average BMI in this sample was 27.47 (standard deviation 4.77).
Recruitment	The UK Biobank recruited approximately 500,000 individuals from 2006 to 2010 with a target age of 40-69 by mailers to people in the UK medical system. Informed consent was obtained by the UK Biobank for all participants.
Ethics oversight	The scientific protocol of the UK Biobank is approved by appropriate external ethics committees in accordance with guidance from relevant bodies. Instead of requiring each applicant to obtain separate ethics approval, UK Biobank has sought generic Research Tissue Bank (TB) approval, which covers the large majority of research using the resource.<br>The original approval for the UK Biobank was granted in 2011 by the National Research Ethics Service (NRES) Committee North West - Haydock. The approval was renewed in 2016 and 2021 by the Health Research Authority, North West - Haydock Research Ethics Committee.<br>For additional information, see https://www.ukbiobank.ac.uk/learn-more-about-uk-biobank/about-us/ethics.<br>This research has been conducted using the UK Biobank Resource under Application Numbers 25214, 44108, and 81358.<br>UKBB participants received no compensation.

Note that full information on the approval of the study protocol must also be provided in the manuscript.

Field-specific reporting

Please select the one below that is the best fit for your research. If you are not sure, read the appropriate sections before making your selection.

☒ Life sciences ☐ Behavioural & social sciences ☐ Ecological, evolutionary & environmental sciences

For a reference copy of the document with all sections, see nature.com/documents/nr-reporting-summary-flat.pdf

Life sciences study design

All studies must disclose on these points even when the disclosure is negative.

Sample size	Sample sizes were determined from the data. We did not pre-define sample sizes based on power estimates. The sample size for each

Sample size	phenotype in this study was determined by the number of individuals which had both complete (i.e., non-missing) phenotype and covariate data. Because of varying levels of missingness for the different phenotypes, the sample size ranged from 406478 to 468386 samples for quantitative traits. Binary traits were extracted using the definitions by Jurgens et al. 2022, Supp. Table 1 (https://doi.org/10.1038/s41588-021-01011-w) and considered as cases if they had a matching code according to the trait definitions. All remaining samples were considered as controls (if not matching an 'exclude' code).
Data exclusions	We removed individuals who had withdrawn consent. To minimize confounding due to population structure and population structure, we restricted to 161,822 unrelated individuals of Caucasian genetic ethnicity as determined by an analysis of genetic principal components for our benchmarking analyses. For biological discovery, we used individuals of all ancestry from the UK Biobank.<br>For binary traits, samples matching an 'exclude' code as defined by the trait definitions were excluded for the respective trait.
Replication	Significant associations for quantitative traits were compared to two studies on a larger cohort from the UK Biobank that employed conventional RVAT strategies (Backman et al., 2021 and Karczewski et al., 2022/Genebass, see 'Data'). For binary traits, we also compared to Jurgens et al., 2022. The replication rate of identified gene-trait associations is shown in Fig. 2c,g for quantitative traits. For binary traits, replication assessment was complicated by variable phenotype definitions across studies and is provided in Supp. Table 9.
Randomization	We did not allocate samples into experimental groups. No randomization was performed.
Blinding	No groups were allocated.

Reporting for specific materials, systems and methods

We require information from authors about some types of materials, experimental systems and methods used in many studies. Here, indicate whether each material, system or method listed is relevant to your study. If you are not sure if a list item applies to your research, read the appropriate section before selecting a response.

Materials & experimental systems

n/a	Involved in the study
☒	☐ Antibodies
☒	☐ Eukaryotic cell lines
☒	☐ Palaeontology and archaeology
☒	☐ Animals and other organisms
☒	☐ Clinical data
☒	☐ Dual use research of concern
☒	☐ Plants

Methods

n/a	Involved in the study
☒	☐ ChIP-seq
☒	☐ Flow cytometry
☒	☐ MRI-based neuroimaging

Plants

Seed stocks	*Report on the source of all seed stocks or other plant material used. If applicable, state the seed stock centre and catalogue number. If plant specimens were collected from the field, describe the collection location, date and sampling procedures.*
Novel plant genotypes	*Describe the methods by which all novel plant genotypes were produced. This includes those generated by transgenic approaches, gene editing, chemical/radiation-based mutagenesis and hybridization. For transgenic lines, describe the transformation method, the number of independent lines analyzed and the generation upon which experiments were performed. For gene-edited lines, describe the editor used, the endogenous sequence targeted for editing, the targeting guide RNA sequence (if applicable) and how the editor was applied.*
Authentication	*Describe any authentication procedures for each seed stock used or novel genotype generated. Describe any experiments used to assess the effect of a mutation and, where applicable, how potential secondary effects (e.g. second site T-DNA insertions, mosiacism, off-target gene editing) were examined.*
